# Newly evolved introns in human retrogenes provide novel insights into their evolutionary roles

**DOI:** 10.1186/1471-2148-12-128

**Published:** 2012-07-28

**Authors:** Li-Fang Kang, Zheng-Lin Zhu, Qian Zhao, Li-Yong Chen, Ze Zhang

**Affiliations:** 1 College of Life Sciences, Chongqing University, Chongqing, 400044, China; 2 Department of Anesthesiology, Research Institute of Surgery, Daping Hospital, Third Military Medical University, 10 Changjiang Zhilu, Chongqing, 400042, China

## Abstract

**Background:**

Retrogenes generally do not contain introns. However, in some instances, retrogenes may recruit internal exonic sequences as introns, which is known as intronization. A retrogene that undergoes intronization is a good model with which to investigate the origin of introns. Nevertheless, previously, only two cases in vertebrates have been reported.

**Results:**

In this study, we systematically screened the human (*Homo sapiens*) genome for retrogenes that evolved introns and analyzed their patterns in structure, expression and origin. In total, we identified nine intron-containing retrogenes. Alignment of pairs of retrogenes and their parents indicated that, in addition to intronization (five cases), retrogenes also may have gained introns by insertion of external sequences into the genes (one case) or reversal of the orientation of transcription (three cases). Interestingly, many intronizations were promoted not by base substitutions but by cryptic splice sites, which were silent in the parental genes but active in the retrogenes. We also observed that the majority of introns generated by intronization did not involve frameshifts.

**Conclusions:**

Intron gains in retrogenes are not as rare as previously thought. Furthermore, diverse mechanisms may lead to intron creation in retrogenes. The activation of cryptic splice sites in the intronization of retrogenes may be triggered by the change of gene structure after retroposition. A high percentage of non-frameshift introns in retrogenes may be because non-frameshift introns do not dramatically affect host proteins. Introns generated by intronization in human retrogenes are generally young, which is consistent with previous findings for *Caenorhabditis elegans*. Our results provide novel insights into the evolutionary role of introns.

## Background

Retroposition, or RNA-based duplication, is the process by which reverse-transcribed mRNAs are inserted into new genomic positions, which generates retrocopies [1]. Retrocopies are assumed not to carry the regulatory regions, but by chance they may obtain functions by recruiting new regulatory elements, and then become functional retrogenes [2-7]. These newly evolved genes may acquire introns in the untranslated regions by capture of nearby exons into a new genomic environment or fusion with host genes, which is chimerization based on intron gain [3-8]. Such retrogenes are usually considered to be intronless because introns were not inherited from the parents. However, in some circumstances, retrogenes may recruit internal exonic sequences as introns [9,10], which is known as intronization [11].

Since intronization of retrogenes was first reported [9], this kind of evolutionary event has been commonly observed in plants. In *Arabidopsis* and *Populus*, 29 retrogenes have undergone intronization, which represent about 15.3 % of all known retrogenes [10]. In contrast, rare cases are reported in vertebrates [12,13]. Previously, only two retrogenes were found to be intronized in mammals [14]. This frequency is extremely low given the thousands of retrocopies in the human (*Homo sapiens*) genome [15-17]. How general retrogene intronization is remains unknown. In the present study, we scanned the human genome for intronized retrogenes and identified nine cases not reported previously. Our results provide new insights into the mechanism of intron gain and expression patterns of retrogenes.

## Methods

### Scanning for intron gain in retrogenes

The human genome data were downloaded from the UCSC Genome Browser database (release hg19) [18,19]. Then, we used the approach of Zhu et al. [10] to search the data for retrocopies. First, we mapped human protein sequences onto the genome with tBLASTn [20] and used the Pseudopipe package [21] to process the raw alignments with the default settings, including tBLASTn *e*-value cutoff (1e-10), coverage cutoff (70 %) and identity cutoff (40 %). Next, we retained candidates with more than three introns absent or only one or two introns absent but with a small *K*_s_ (<2) or other RNA-based duplication evidence, for example, a poly(A) track. Finally, as described previously [10], we set filters to discard possible DNA-based duplication cases. In brief, we discarded all retrocopies in which at least 50 % of the region overlapped with repeats or with flanking genes similar to the parental gene’s flanking regions. We also discarded all retrocopies that aligned well with the introns of the parents. Ultimately, we identified 3436 retrocopies.

We wrote a series of PERL programs to look for intron-containing retrogenes on the basis of annotations from ENSEMBL (GRCh37) [22,23]. We identified 54 candidates of intronized retrogenes for further study.

### Gene structure validation by transcription evidence

We utilized the mRNA and EST annotations from the UCSC Genome Brower Database to search for transcription evidence of intron gain in retrogenes [18,19]. For each sample, we inspected the annotated intronic region to see whether there were transcripts that support its splicing. If transcripts were present, we mapped them on the human genome with BLAT [24] to check whether these transcripts uniquely correspond to the retroposed region. By this method, eight intron-containing retrogenes were validated (Additional files 1 and 2).

### *K*_a_ and *K*_s_ calculation

We estimated the non-synonymous substitution rate (*K*_a_), synonymous substitution rate (*K*_s_) and *K*_a_/*K*_s_ values between the intronic regions of retrogenes and their parental copies, by implementing the codeml program in the PAML package following the Nei-Gojobori method [25,26] and analyzed the results with the likelihood ratio test. We did *K*_a_/*K*_s_ estimation between the exonic regions of retrogenes and their parental copies in the same way.

### RT-PCR

In order to validate the structure of the retrogenes, we collected samples of 16 human tissues from Daping Hospital, Chongqing, for experiments (Additional file 3). Following the manufacturer’s instructions, we used TRIzol Reagent (Invitrogen, Carlsbad, CA) to isolate RNA and digested the contaminating genomic DNA with RNase-free DNase I (Promega, Madison, WI). cDNAs were synthesized with Moloney murine leukemia virus reverse transcriptase (Promega). We performed PCR in a 25 μl reaction volume, and 5 μl of the PCR products were electrophoresed on a 1.2 % agarose gel. To validate whether the smaller-sized bands represented the retrogenes, we cloned and sequenced those PCR products. Ultimately, we identified two samples in which the sequences of the smaller-sized bands belonged to retrocopies and the larger bands to the parental genes (Additional file 4).

### Peptide support for intronized retrogenes

To identify whether one retrogene was expressed at the protein level, we sought peptide evidence in the PeptideAtlas [27-29] and PRIDE [30,31] databases using the gene name. Each search result displayed experimental details including the fractionation and sequencing (by mass spectroscopy or other methods) of short peptides. Among the results, we extracted peptides that matched the protein sequence of the intronized retrogene. Given that one peptide may match many proteins, we also used BLASTp [32,33] to ensure that the peptide specifically mapped to the gene we targeted. We only retained peptides for which the best hit was a targeted protein.

### Age estimation of the retrogenes

We examined the presence and absence of orthologs in the phylogenetic tree for vertebrates and used the established origination times of all human genes [34] to infer the times of origin of the retrogenes. For comparison we used the same method to estimate the time of origin of 27 retrogenes that recruited introns by chimerization [8]. We mapped the results on the vertebrate phylogeny (Additional files 5,6 and 7). The timeline and divergence time of species in the phylogeny were reconstructed based on data from the UCSC Genome Browser database and other sources [19,34-40].

### Detection of splicing signals

We detected splicing signals of new introns with SROOGLE [41]. For an intron X, if its upstream exon is Y and downstream exon is Z, we used X and Y to detect signals of the 5′ splice site (SS) and X and Z for that of the branch site (BS), polypyrimidine tract (PPT), and 3′ SS. We performed two detections for each intron; one was performed on the parental gene and the other was done for the retroposed sequence. The former and latter were considered to represent the status before retroposition and the current status, respectively. Finally, for each detection, we recorded the percentile score for constitutive introns, which was obtained from a data set composed of >50,000 constitutive introns [41], because all introns in our data set showed no evidence for alternative splicing (Additional file 8).

## Results

### Identification of intron gain in retrogenes

We focused on identifying retrogenes that contain introns and scanned the human genome using a published pipeline [10]. We mapped all human proteins onto the genome with tBLASTn [20] and extracted all possible candidates of retrocopies from among the results with PseudoPipe [21]. Then, we set filters to exclude cases that did not fulfill the properties of retroposition and obtained 3436 retrocopies. Finally, we determined that 54 of the 3436 retrocopies contained introns on the basis of gene structure annotations from ENSEMBL [22,23].

We used two methods to validate the existence of retrogene introns. First, we collected information from the UCSC Genome Browser database [18,19] and found eight cases with confident transcriptional evidence (Additional file 2). Next, we performed experiments to validate the existence of the retrogene introns. Given the high similarity in the flanking regions of new introns for most retro-parental alignments, we designed pairs of primers whose products (Additional file 9) spanned the intronic regions for both the retrogenes and their parental genes. Theoretically, the amplified segments from the retrogenes (without the intronic sequences) would be smaller than those of the parental genes (with the intronic sequences). By this method, we confirmed that two retrogenes contained introns (Additional file 4), one of which was one of the eight retrogenes mentioned above. In total, we identified nine retrogenes that evolved introns in the retroposed regions (Table 1). Our data did not include RNF113B and DCAF12, which were reported in a previous study [14], because the parents of these two retrogenes were lost after the divergence of mammals from vertebrates, whereas our pipeline used parental protein sequences as queries to search for retrocopies. In addition, we discarded POM121L2 and ARPM1, which were suggested to be intronized retrogenes previously [8], because the alignment identities of these retrogenes and their respective parents did not fulfill the criteria set in our pipeline (>40 % identity).

**Table 1:** Nine human retrogenes that gained introns investigated in this study

**Retrogene**	**Parent**	**Movement**	**Intron (+)**		**Intron (−)**	**Evidence**
TMEM14D	TMEM14B	10 < −6	1		4	A
RPS3AP5	RPS3A	10 < −4	1		5	B
XXyac-R12DG2.2	RCN1	13 < −11	2*		5	B
HSP90B2P	HSP90B1	15 < −12	2		16	B
HSP90AA4P	HSP90AA1	4 < −14	3		9	A,B
HSP90AA5P	HSP90AA1	3 < −14	2		7	B
CSMD3	RPL18	8 < −19	1		5	B
WBP2NL	SLC25A5	22 < −X	1		3	B
AC019016.1	CSNK1A1	15 < −5	2*		8	B

In the column ‘Movement’, ‘10 < −6’ means a new gene on chromosome 10 is retroposed from a gene on chromosome 6, for example. ‘Intron (−)’ and ‘Intron (+)’ are the numbers of intron losses and intron gains in retrocopies, respectively. For ‘Evidence’, ‘A’, confirmed by RT-PCR; ‘B’, supported by convincing transcription evidence. ‘*’ means that the newly evolved intronic regions of XXyac-R12DG2.2 and AC019016.1 could be spliced in two patterns, respectively.

### Mechanisms of intron gain in retrogenes

**Figure 1:**
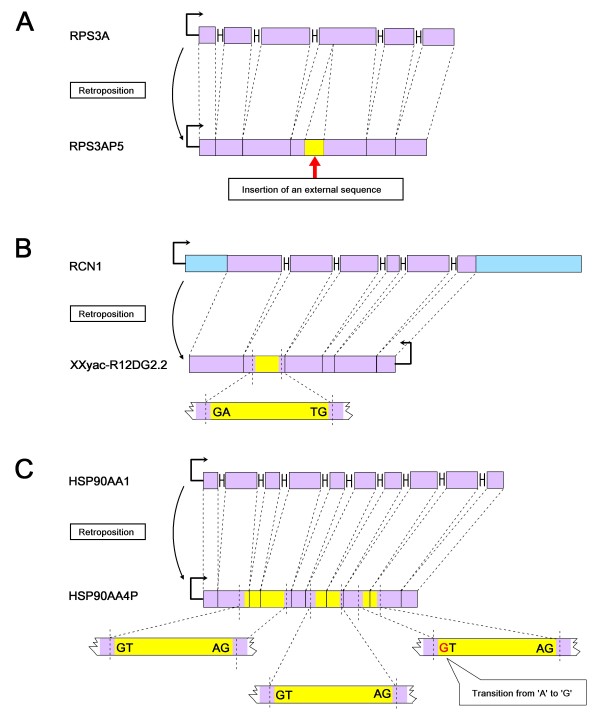
**Mechanisms of intron gain in retrogenes.** In the parental gene, rectangles represent exons, ‘H’-like tags represent introns, the retroposed regions are indicated in purple, and other regions are indicated in blue. In the retrogene, the retroposed region is indicated in purple and the newly evolved intronic regions are indicated in yellow. Semi-rectangle lines with arrows indicate the direction of transcription. **(A)** The retrogene RPS3AP5 gained an intron by insertion of an external sequence; **(B)** the retrogene XXyac-R12DG2.2 evolved a new intron after transcription in the opposite orientation compared to the parent; **(C)** in retrogene HSP90AA4P three new introns were generated by intronization. There is no mutation at the splice sites in the two introns near the 5′ terminus, whereas one transition from ‘A’ to ‘G’ (indicated in red) at the splice sites occurred in the intron near the 3′ terminus.

To clarify the intron-gain mechanisms of these retrogenes, we produced protein and nucleotide sequence alignments for the retrogenes and their respective parental genes (Additional files 10 and 11). For RPS3AP5, we observed that its intronic region did not have counterparts in the parental gene. This result indicated that this retrogene did not gain the intron by intronization, but rather by insertion of an external sequence (Figure 1A). Using the inserted sequence as a query for a BLAT [24] search against the human genome, we identified more than five paralogous sequences with identity >95 % and coverage >70 %. The new intron may be derived from one of these paralogs. By checking the genome annotations in the UCSC Genome Browser database [18,19], we found that none of these paralogs were annotated as introns. Thus, the new intron may not have originated by ‘reverse splicing’, the process by which a spliced-out intronic RNA is inserted into a novel site of one RNA gene transcript by reversal of the splicing reaction [12,42]. The intron may have been created by a mechanism not reported previously.

We observed that three retrogenes (XXyac-R12DG2.2, CSMD3 and WBP2NL) were transcribed in the reverse direction relative to that of their parents. For XXyac-R12DG2.2 there are 10 annotated transcription patterns and introns appeared in four of the 10 patterns (Additional file 2). Taking ENST00000379050 as an example, the retrocopy contained a 170 bp intron, and its splicing donor and acceptor sites (‘GT’ and ‘AG’) had reverse counterparts (‘AC’ and ‘AT’) in the parental gene (Figure 1B, Additional files 10 and 11). Thus, transcription in the reverse orientation led to the origin of the intron splicing sites. For the remaining three transcription patterns (ENST00000522673, ENST00000519494 and ENST00000330825), the newly evolved intron was shorter (127 bp) and the retroposed sequence was located near the 3′ end. In addition, the retrocopy is inserted near the 3′ end of a ncRNA gene candidate (LOC 100190939, Additional file 12).

In CSMD3, the retroposed region was located at the 5′ untranslated region (UTR) of the mRNA. Some part of the retrocopy had changed into an intergenic sequence, and some part acted as a portion of an intron (Additional files 2 and 12). The retrogene was located in the first intron of WBP2NL (Additional file 12). Nevertheless, the retrocopy might be transcribed at least some of the time, because an mRNA sequence, BC03789, supports the transcription of this retrogene (Additional file 1 and 2). We did not find evidence for protein-level expression of the three retrogenes that gained an intron after transcription in the reverse orientation. The new introns in these three retrogenes were annotated to be in non-coding regions.

The remaining five retrogenes had gained introns through intronization, which generated 10 new introns. Taking HSP90AA4P as an example, three exonic sequences were changed into introns (Figure 1C). Eight of the 10 introns had the canonical splicing boundaries ‘GT-AG’. 80 % (8/10) of the introns arose in ORF and 20 % (2/10) in UTRs.

In total, we observed three mechanisms of intron gain for these retrogenes. In addition to intronization, retrogenes may gain introns after insertion of external sequences or transcription in the opposite orientation compared to the parent (Figure 1).

### Non-frameshift introns generated by intronization had greater evolutionary success

For the five retrogenes that underwent intronization, we examined the alignments of retrocopies and their corresponding parental sequences to assess whether these introns had disturbed the frame of putative translation inherited from the parental genes (Additional file 11). If one intron disturbed the frame, we termed it a frameshift intron, otherwise it was considered to be a non-frameshift intron. The lengths of the corresponding sequences of the five retrogenes (70 %) were in multiples of three bases. We performed a manual check for each retrogene. At the location 100 bp upstream of the second intron of HSP90AA4P (from 5′ to 3′, HSP90AA4P-2), we observed an insertion of 23 bases. The length of HSP90AA4P-2 was 83 bp. Thus, compared with the parent, the intron and insertion led to an overall loss of 60 bases (divisible by three) in the transcript. Similarly, for HSP90AA5P we observed an insertion of 22 bases located 1 bp upstream of the intron near the 5′ terminus (HSP90AA5P-1) and a deletion of four bases located 2 bp upstream of the intron near the 3′ terminus (HSP90AA5P-2). The lengths of these two introns were 439 and 254 bp, respectively. As in HSP90AA4P-2, both the indels and intronization shortened the coding sequences by 417 and 258 bp in HSP90AA5P-1 and HSP90AA5P-2, respectively (both numbers are divisible by three). Both were classified as non-frameshift introns. The two alternative spliced introns of AC019016.1 were annotated to be UTR-region introns according to the UCSC database [18,19] and Ensembl [22,23].

In total, eight of the 10 introns created by intronization were non-frameshift introns. This proportion (80 %) is significantly higher than the percentage of frameshift introns generated by chimerization based on intron-gain retrogenes (29.8 %, 16/49) (*P*-value = 0.017) [8]. From searches of PeptideAtlas [27-29] and PRIDE [30,31], we found that the predicted proteins of HSP90B2P, HSP90AA4P and HSP90AA5P had respective unique matching peptides (Table 2), which indicated the true protein-coding activity of these transcripts. Consistent with findings for *Caenorhabditis elegans*[11], our observations showed that non-frameshift introns had greater evolutionary success.

**Table 2:** Peptide support for intronized retrogenes

**Gene name**	**Peptide match**	**Peptide database reference**^**a**^	**Location in protein seq**	**BLASTP hits**^**b**^
HSP90B2P	NLNFVKGVVDSGGLSLNVSCETLQQHK	PRIDE: 8670	86	Self (4e-19, 100 %)
	IEKAMVSQCLTESLCALVASQYGWSGNMER	PRIDE: 8670	270	Self (4e-24, 100 %)
	AMVSQCLTESLCALVASQYGWSGNMER	PRIDE: 8671; 8668	273	Self (7e-21, 100 %)
	MAETIQEVEDEYKAFCK	PRIDE: 8672	1	Self (9e-11, 100 %)
	CVFITDDFRDTMPK	PRIDE: 8669	72	Self (7e-08, 100 %)
HSP90AA4P	HNNDEQYAWESSLR	PeptideAtlas: PAp00393519	93	Self (1e-07, 100 %)
	ADLINNLGTITK	PeptideAtlas: PAp01587648	20	Self (8e-04, 100 %)
	DQVANSTIVQR	PeptideAtlas: PAp00565957	207	Self (0.005, 100 %)
HSP90AA5P	IKEIVKKHSQFIGYPITLFVEKKR	PeptideAtlas: PAp00040955; PAp00423980	33	Self (2e-17, 100 %)
	HGLEVIYMIELIDKYCVQQLK	PeptideAtlas: PAp00040711	199	Self (2e-15, 100 %)

‘a’, Database name and experiment numbers or identifiers. ‘b’, BLASTP search against the GenBank non-redundant protein database (e-value and maximum identity of the match are shown in parentheses [32,33]).

### Retrogenes underwent intronization by cryptic splicing sites

**Figure 2:**
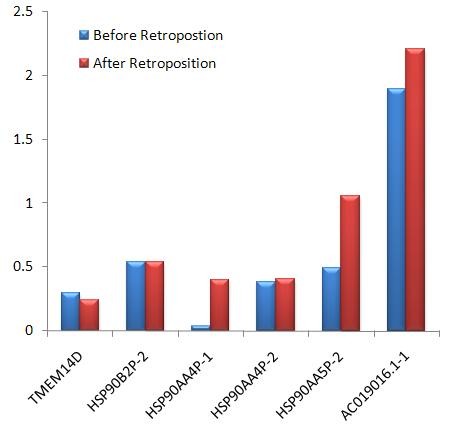
**Comparison of splicing signals of retrogene introns (after retroposition) and their corresponding regions in the parental gene (before retroposition).** The y-axis is the sum of the percentile scores of four different signals, comprising the branch site (BS), polypyrimidine tract (PPT), and 5′ and 3′ splice sites (Table 3). The higher the score, the stronger the splicing signal. Scores for BS and PPT were calculated with algorithm ‘K’ [47] and those for the splice sites were calculated with ‘M’ [48] by SROOGLE [41]. Six different retrogene introns are plotted on the x-axis. If a retrogene evolved only one intron (TMEM14D), we used the gene name to represent the intron, or we marked different introns in one retrogene in the format of the gene name plus a serial number following the hyphen. For example, ‘HSP90AA4P-1’ represents the first intron (in the direction from 5′ to 3′) in the retrogene HSP90AA4.

Previous studies showed that most intronizations were caused by base substitutions at the 5′ and 3′ SS [10,11]. However, we observed only four such cases (40 % of all cases) in our data set. By inspecting the EST annotations for the corresponding parental regions of all newly intronized introns, we found that none of these intronized introns was created by inheriting alternative splicing sites from the parental gene. What led to the creation of the other six retrogene introns? Since a retrogene does not contain introns compared with its parental gene, we proposed that the new introns were created by cryptic splice sites in the exonic regions of the parents. That is, cryptic splice sites were silent in the parents, but were activated in the retrogenes after retroposition and the new introns were generated. To test our hypothesis, we used SROOGLE [41] to detect the splicing signals (5′ SS, 3′ SS, the PPT located upstream of the 3′ SS, and the BS located upstream of the PPT) of the retrogene introns and their respective corresponding regions in the parental genes. The splicing signals of introns in four of the six retrogenes were increased, except for those of TMEM14D and HSP90B2P (Figure 2, Table 3). For the latter two retrogenes, in the parental gene the corresponding regions of the retrogene introns had lower splicing signals compared with those of neighboring introns (Additional file 13 and Additional file 14). It is likely that these cryptic intronic regions were oppressed in the parental genomes and the oppression was released after retroposition. The splice sites of these six new introns pre-existed but were cryptic in the parental genes. After retroposition, the splice sites were activated in the novel genomic environments. In addition, for the four introns that showed base substitutions at their splice sites, the splicing signals increased not only at the 5′ SS and 3′ SS but also at the BS and PPT (Table 3). In addition to point mutation, the change in gene structure after retroposition might also contribute to the evolution of new introns.

**Table 3:** **Percentile scores **[41]** of splicing signals of retrogene introns (after retroposition) and their corresponding regions in the parental gene (before retroposition)**

**Intron symbol**	**Splice sites**	**After retroposition**				**Before retroposition**			
		**BS (K)**	**PPT (K)**	**5′ SS (M)**	**3′ SS (M)**	**BS (K)**	**PPT (K)**	**5′ SS (M)**	**3′ SS (M)**
TMEM14D	GC-AG	0.14	0.06	0.02	0.02	0.14	0.04	0.01	0.11
(HSP90B2P-1)	GT-AG	0.39	0.39	0	0.01	0.39	0.24	0	0
HSP90B2P-2	GT-AG	0.5	0.03	0	0.01	0.5	0.02	0	0.02
HSP90AA4P-1	GT-AG	0.21	0.03	0.04	0.12	0	0	0.04	0
HSP90AA4P-2	GT-AG	0.03	0.06	0.04	0.28	0.03	0.06	0.04	0.25
(HSP90AA4P-3)	GT-AG	0.56	0.33	0	0.03	0.56	0.22	0	0.02
(HSP90AA5P-1)	TT-AG	0.25	0.27	0	0.54	0	0	0	0
HSP90AA5P-2	GT-AG	0.45	0.45	0.01	0.15	0.12	0.15	0.01	0.21
AC019016.1-1	GT-AG	0.91	0.35	0.11	0.84	0.61	0.35	0.11	0.82
(AC019016.1-2)	GT-AG	0.91	0.35	0.47	0.84	0.61	0.35	0	0.82

The higher the score, the stronger the splicing signal is. The scores for BS and PPT were calculated with the ‘K’ algorithms [47], and those for 5′ SS and 3′ SS were calculated with ‘M’ [48] by SROOGLE [41]. The intron symbol is in the format of the gene name plus a serial number following the hyphen. For example, ‘HSP90B2P-1’ indicates the first intron (in the direction from 5′ to 3′) in HSP90B2P. If a retrogene evolved only one intron (TMEM14D), the intron is represented by the gene name. In the column ‘Intron symbol’, parentheses indicate that the splice sites underwent base substitution.

### Intronization tended to occur in young retrogenes

In *C. elegans*, intronization is reported to be a major contributor to intron creation and most introns generated by this mechanism are young [11]. In our data set, 66.7 % of retrogene introns (10/15) were created by intronization. This finding is consistent with previous studies [11]. We used the established origination times of all human genes to trace the time of origin of intronized retrogenes [34] and examined the presence and absence of the corresponding orthologs in the vertebrates phylogeny (Additional file 6). We found that 80 % (4/5) of the intronized retrogenes were primate specific. We also recalculated the ages of 27 chimerizations based on intronized retrogenes with the same method [8] (Additional file 7) and found that only 18.5 % of intronized retrogenes (5/27) were primate specific. This finding indicated that intronization tended to occur in young retrogenes (proportion test, *P* = 0.023). Furthermore, in our data set, no intronized retrogene (0/5) was retroposed from chromosome X (‘out-of-X’). The retrogenes from chromosome X were mostly old and evolved after the divergence of eutherian mammals (human or mouse) and marsupials (opossum) [34]. For retrogenes that underwent intron gains by chimerization, the proportion of ‘out-of-X’ retrogenes was 37 % (Additional file 7). Therefore, the comparison of 0 % and 37 % reinforced the conclusion that intronization tended to occur in young retrogenes.

### Evolutionary rates of intronized retrogenes

To evaluate the evolutionary rates of retrogenes, we calculated *K*_a_, *K*_s_, and *K*_a_/*K*_s_ values between the intronic regions of retrogenes and their parental copies as well as between the exonic regions of retrogenes and their parental copies. The *K*_a_ values in the intronic regions were higher than those in the exonic regions (Mean_intronic_ = 0.207, Mean_exonic_ = 0.111, Wilcoxon two-sample test, *P*-value = 0.098; Table 4). Similarly, *K*_s_ values in the intronic regions were higher than those in the exonic regions (Mean_intronic_ = 0.263, Mean_exonic_ = 0.151, Wilcoxon two-sample test, *P*-value = 0.194). These findings are consistent with the conclusion that introns evolved faster than exons.

**Table 4:** Substitution rates between the intronic and exonic regions of retrogenes and their corresponding regions of parental genes

**Retrogene**	**Intronic region**					**Exonic region**				
	***K***_**a**_	***K***_**s**_	***K***_**a**_**/*K***_**s**_	***P*-value**	**Length**	***K***_**a**_	***K***_**s**_	***K***_**a**_**/*K***_**s**_	***P*-value**	**Length**
TMEM14D^c^	0.062	0.058	1.074	0.936	105	0.006	0.014	0.440	0.570	237
RPS3AP5^a^	NA	NA	NA	NA	NA	0.017	0.014	1.210	0.172	780
XXyac-R12DG2.2^b^	0.024	0.029	0.830	0.892	129	0.008	0.012	0.643	0.631	813
HSP90B2Pa^c,*^	0.823	0.597	1.379	0.526	144	0.045	0.067	0.678	0.091	2163
HSP90AA4P^c,*^	0.104	0.277	0.374	0.000	744	0.055	0.085	0.656	0.000	1374
HSP90AA5P^c,*^	0.087	0.215	0.406	0.001	672	0.088	0.221	0.400	0.082	897
CSMD3^b^	0.313	0.575	0.544	0.051	291	0.186	0.282	0.659	0.310	225
WBP2NL^b^	0.033	0.088	0.373	0.192	177	0.385	0.377	1.021	0.919	684
AC019016.1^c^	0.083	0.082	1.010	0.978	636	0.081	0.175	0.466	0.084	273

*K*_a_ represents the non-synonymous substitution rate and *K*_s_ indicates the synonymous substitution rate. The *P*-value was calculated with the likelihood ratio test and the null hypothesis was *K*_a_/*K*_s_ =1. NA: not available (the corresponding parental sequence of the new intron in retrogene RPS3AP5 did not exist, because the intron was created by insertion of an external sequence). ‘a’, The retrogene gained introns by insertion of an external sequence. ‘b’, The retrogene gained introns after transcription in the opposite orientation compared to the parent. ‘c’, The retrogene gained introns by intronization. ‘*’, Evidence at the protein level for transcription of the retrogene was obtained.

In addition, the exonic regions of most intronized retrogenes had *K*_a_/*K*_s_ values smaller than 1 (*P*-value < 0.1), which suggested that the corresponding regions were under negative selection. By checking for evidence of expression, we found that three of the five intronized retrogenes showed evidence for expression at the protein level and the additional two retrogenes showed transcription evidence at the RNA level. This result indicated that most intronized retrogenes were functional and should be under negative selection.

With regard to the three retrogenes that gained introns after transcription in the opposite orientation compared with the parent, they were annotated to be in the non-coding regions of other genes. We observed that CSMD3 and WBP2NL evolved faster than the other retrogenes (Table 4). This finding is consistent with the conclusion that non-coding regions such as UTR regions are under less functional constraint than coding regions. However, XXyac-R12DG2.2 evolved slowly relative to that of CSMD3 and WBP2NL. Thus, XXyac-R12DG2.2 is likely to be under functional constraint.

## Discussion

In this study, we systematically searched the human genome for retrogenes that underwent intron gain in the coding region and in total identified 15 retrogene introns. These newly generated introns evolved at a faster rate than neighboring exons. In contrast to the findings in plants [10], we found that intron gain events in retrogenes were rare in humans. In spite of this rarity, the mechanisms of intron creation in these retrogenes are diverse. We found that retrogenes could gain introns in three ways: insertion from an external sequence, transcription in the opposite direction compared with the parent, and intronization. For the latter method, in addition to base substitution, retrogenes also may create introns in exonic regions via cryptic splice sites, which might be activated by the new gene structure after retroposition. Consistent with the findings in *C. elegans*[11], retrogene introns generated by intronization in humans are generally young and are mostly located in the coding region of the new gene. The retrogenes that underwent intronization in coding regions all retained the parental frames of translation and most showed expression evidence at the protein level. The significantly higher percentage of non-frameshift introns implied that this kind of intron possessed a higher likelihood of persistence after intronization. The reason for this may be that frameshift introns mostly have a major effect on the proteins. Thus, non-frameshift introns are more likely to survive. However, non-frameshift introns may be neutral in effect, as proposed previously [43,44]. Furthermore, previous studies have shown that the rate of intron loss is much larger than that of intron gain in mammals [12,13,45]. Consequently, the older the retrogene is, the more probable the retrogene will lose the intronized exon, and this may explain why such introns are mainly observed in young retrogenes.

Some questions arise from careful examination of our observations. For example, for the retrogene RPS3AP5, in which the new intron was created by insertion of an external sequence, the process by which the new intron was created is unknown. In addition, in searches of UCSC [18,19], Ensembl [22,23], PeptideAtlas [27-29] and PRIDE [30,31], we did not obtain evidence of protein-level expression for the three retrogenes that gained introns after transcription in the reverse orientation compared with their parents. The new introns in these three retrogenes were annotated to be in non-coding regions and appeared to be parts of existing intron-containing genes, as described previously [7]. Thus, these retrogenes generally evolved faster than intronized retrogenes (Table 4).

For the eight non-frameshift introns generated by intronization, we examined whether they are under natural selection by checking their genetic variation in different human populations with the 1000 Genomes Browser [46]. However, we did not find insertions, deletions or mutations in splice sites in seven of these retrogenes (Additional file 14), which implied that they are nearly fixed in all populations and may be under negative selection. In addition, there is a possibility that this pattern observed was caused by genetic drift because generation of new introns may be neutral. Finally, what is the importance of producing a shorter protein than the protein from the parent gene? This question may be answered by comparing the functions of the original proteins and that encoded by the retrogenes in the future.

## Conclusions

Our results showed that retrogenes may gain introns in three ways: insertion from an external sequence, transcription in the reverse direction compared to that in the parent, and intronization. In addition to base substitution, intronization also may be promoted by cryptic splice sites. For introns generated by intronization, non-frameshift introns might have greater evolutionary success than frameshift introns, because non-frameshift introns have only a small effect on the host proteins or are neutral. Furthermore, intronization tended to occur in young retrogenes.

## Abbreviations

BS, branch site; PPT, polypyrimidine tract; SS, splice site.

## Competing interests

The authors declare that they have no competing interest.

## Authors’ contributions

LFK and ZLZ together carried out the identification of intronized retrogenes and data analysis, and performed the statistical analyses. LFK performed the PCR analysis and helped to draft the manuscript. ZLZ conceived the study, participated in its design and analysis, and drafted the manuscript. QZ helped to perform the data analysis and statistical analyses, participated in the design of the study and helped to draft the manuscript. LYC provided the materials for experiments. ZZ participated in the design of the study and helped to draft the manuscript. All authors read and approved the final manuscript.

## Supplementary Material

Additional file 1 **Transcripts uniquely mapped to retrogenes.** This file lists transcripts that spanned the introns of their mapped retrogenes Click here for file

Additional file 2 **Evidence for transcription of retrogene introns (from the UCSC Genome Browser database).** This file contains snapshots from the UCSC Genome Browser database that displays the transcription of retrogenes that gained introns Click here for file

Additional file 3 **List of human tissues sampled for the experiments.** This file lists the human tissues that we used for the experiments to validate the existence of retrogene introns Click here for file

Additional file 4 **Experimental validation of retrogene introns in TMEM14D and HSP90AA4P.** This file shows the experimental results for validating the existence of retrogene introns Click here for file

Additional file 5 **Phylogenetic tree for vertebrates.** A diagram of the phylogenetic tree for vertebrates Click here for file

Additional file 6 **Chromosome and time of origin of intronized retrogenes.** This file shows the origination times of intronized retrogenes Click here for file

Additional file 7 **Chromosome and time of origin of retrogenes that gained introns by chimerization.** This file shows the origination times of retrogenes that gained introns by chimerization Click here for file

Additional file 8 **Transcription annotations (from the UCSC Genome Browser database) of retrogene introns in the parental gene.** This file contains snapshots from the UCSC Genome Browser database displaying transcription annotations of retrogene introns in the parental gene Click here for file

Additional file 9 **Sequences of primer pairs used to amplify the retrogenes and their parents.** A table that lists primer pairs we used to amplify the retrogenes and their parents Click here for file

Additional file 10 **Protein-level alignments of intron-gain retrogenes (“Sbjct”) and their parents (“Query”) by GeneWise.** This file contains alignments of intron-gain retrogenes and their parents in protein level Click here for file

Additional file 11 **Nucleotide-level alignments of retrogene introns (‘Sbjct’, blue and red, splice sites) and parental genes (‘Query’, program NCBI-BLAST).** This file contains alignments of intron-gain retrogens and their parents in DNA level Click here for file

Additional file 12 **Positions of three retrogenes (XXyac-R12DG2.2, CSMD3 and WBP2NL) in the human genome (from the UCSC Genome Browser database).** This file contains snapshots from the UCSC Genome Browser Database displaying the positions of three retrogenes Click here for file

Additional file 13 **Comparison of splicing signals (percentile score) in the corresponding region of the new intron in the parental gene and neighboring introns.** This file shows the results for the comparison of splicing signals in the corresponding region of the new intron in the parental gene and neighboring introns Click here for file

Additional file 14 **Genetic variation of four retrogenes in different human populations.** This file displays alignments of genomes of different human populations in the region of four retrogenes. Click here for file
