# Supplementary material for: Newly evolved introns in human retrogenes provide novel insights into their evolutionary roles
Source: BMC Evol Biol. 2012 Jul 28;12:128. doi: 10.1186/1471-2148-12-128 (PMC3565874; doi:10.1186/1471-2148-12-128)
Supplement: Additional file 1 — Transcripts uniquely mapped to retrogenes. This file lists transcripts that spanned the introns of their mapped retrogenes [file 1471-2148-12-128-S1.doc]

**Additional file 1**

**Transcripts uniquely mapped to retrogenes.**

| Gene Name | Transcripts |
| --- | --- |
| RPS3AP5 | BG827133, BQ304679, BG180066, BF088835, EC488010, EC529310, BQ318052, EC500271, EC444641 |
| XXyac-R12DG2.2 | BM702699, AL538339, CB105370, AA402745, BU627860, AL524734, AA701879, AL538338, AI248633, AI241463 and so on (number of mapped transcripts>10) |
| HSP90B2P | AY956768 |
| HSP90AA4P | AY956760 |
| HSP90AA5P | AY956761 |
| CSMD3 | AB114605 |
| WBP2NL | BC038789 , BI461473 |
| AC019016.1 | BC028192, DB527361, DB518216, BI825331, BI831584, BC066972, BG723778 |

Listed transcripts all spanned the introns of their mapped retrogenes. For XXyac-R12DG2.2, there are 11 annotated transcription patterns, of which 4 are involved to intron gain in the retroposed region (ENST00000379050, ENST00000522673, ENST00000519494 and ENST00000330825). The existence of the new intron in ENST00000379050 is supported by BM702699, while that in ENST00000522673, ENST00000519494 and ENST00000330825 are supported by other transcripts. AC019016.1 transcribed in two ways, CSNK1A1P (UCSC Gene Name; [S1, S2]) and ENST00000430593. The gene structures of these two transcription patterns are supported by BC028192, DB527361, DB518216, BI825331 and BC066972, BG723778, separately.

**References**

S1. Karolchik D, Hinrichs AS, Furey TS, Roskin KM, Sugnet CW, Haussler D, Kent WJ: **The UCSC Table Browser data retrieval tool.** *Nucleic Acids Res* 2004, **32(Database issue)**:D493-496.

S2. Kuhn RM, Karolchik D, Zweig AS, Wang T, Smith KE, Rosenbloom KR, Rhead B, Raney BJ, Pohl A, Pheasant M, Meyer L, Hsu F, Hinrichs AS, Harte RA, Giardine B, Fujita P, Diekhans M, Dreszer T, Clawson H, Barber GP, Haussler D, Kent WJ: **The UCSC Genome Browser Database: update 2009**. *Nucleic Acids Res* 2009, **37(Database issue)**:D755-761.
