# Supplementary material for: Newly evolved introns in human retrogenes provide novel insights into their evolutionary roles
Source: BMC Evol Biol. 2012 Jul 28;12:128. doi: 10.1186/1471-2148-12-128 (PMC3565874; doi:10.1186/1471-2148-12-128)
Supplement: Additional file 2 — Evidence for transcription of retrogene introns (from the UCSC Genome Browser database). This file contains snapshots from the UCSC Genome Browser database that displays the transcription of retrogenes that gained introns [file 1471-2148-12-128-S2.doc]

**Additional file 2**

**Evidence for transcription of retrogene introns (from the UCSC Genome Browser database [S1, S2]).**

Contents in parentheses are the ENSEMBL ID (e.g. ENST00000430593) or UCSC Gene Name (e.g. CSNK1A1P) of transcription patterns (for XXyac-R12DG2.2, we only listed the ones that contain introns in the retrocopy).

**References**

S1. Karolchik D, Hinrichs AS, Furey TS, Roskin KM, Sugnet CW, Haussler D, Kent WJ: **The UCSC Table Browser data retrieval tool.** *Nucleic Acids Res* 2004, **32(Database issue)**:D493-496.

S2. Kuhn RM, Karolchik D, Zweig AS, Wang T, Smith KE, Rosenbloom KR, Rhead B, Raney BJ, Pohl A, Pheasant M, Meyer L, Hsu F, Hinrichs AS, Harte RA, Giardine B, Fujita P, Diekhans M, Dreszer T, Clawson H, Barber GP, Haussler D, Kent WJ: **The UCSC Genome Browser Database: update 2009**. *Nucleic Acids Res* 2009, **37(Database issue)**:D755-761.

**RPS3AP5 (ENST00000389400)**

**
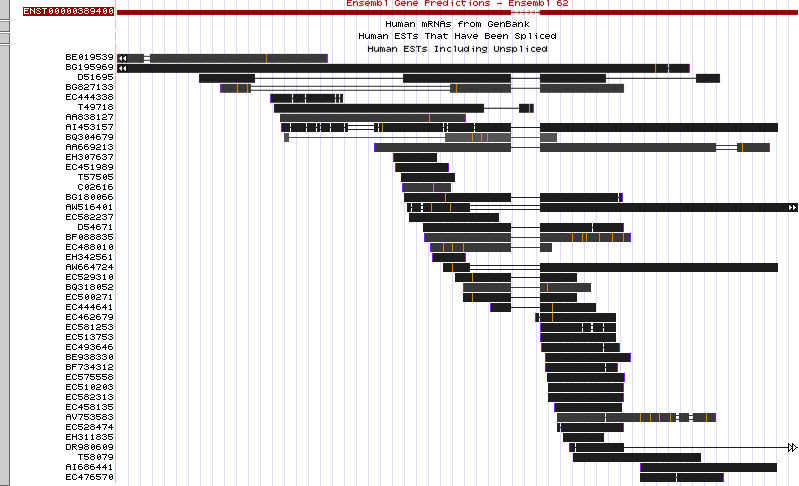
**

**XXyac-R12DG2.2 (ENST00000379050, ENST00000522673, ENST00000519494 and ENST00000330825)**

**
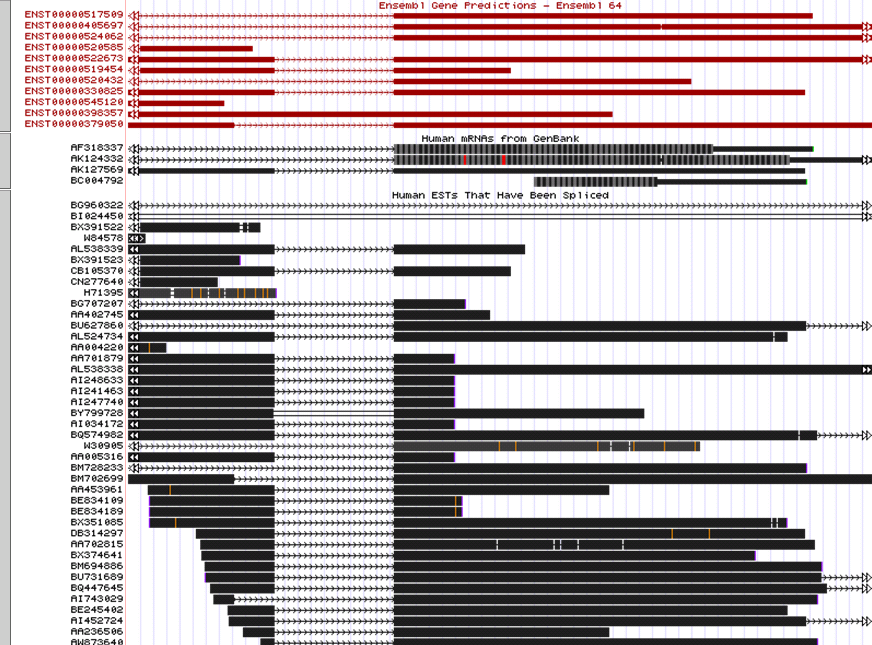
**

**HSP90B2P (ENST00000378906)**

**
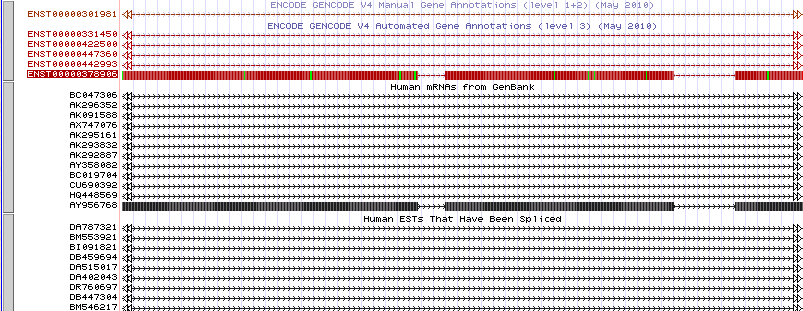
**

**HSP90AA4P (ENST00000378770)**

**
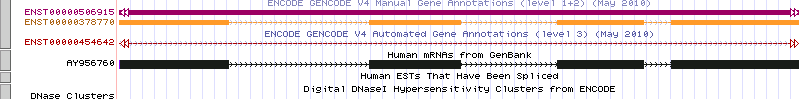
**

**HSP90AA5P (ENST00000382482)**

**
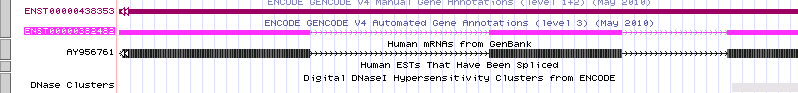
**

**CSMD3 (ENST00000343508)**

**
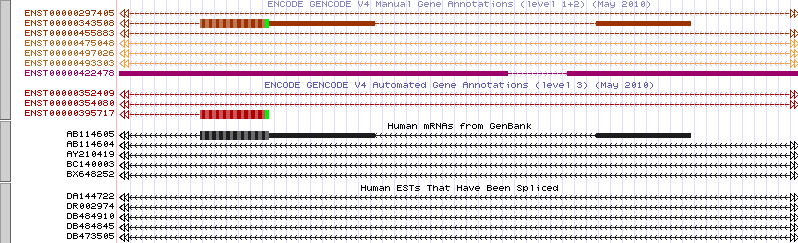
**

**WBP2NL (ENST00000461730)**

**
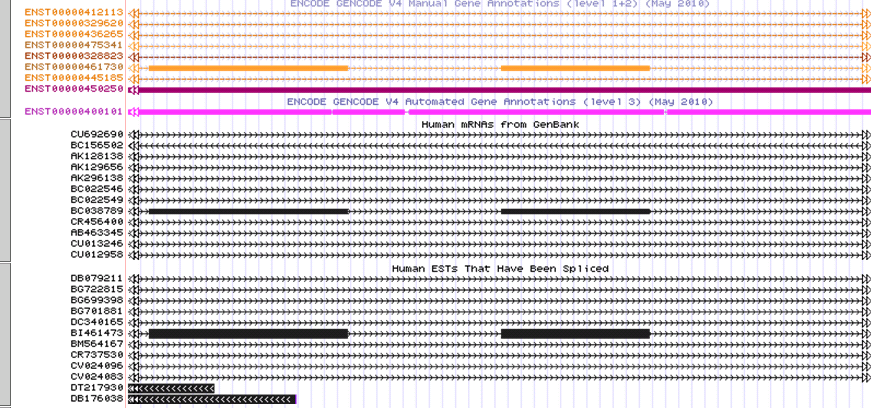
**

**AC019016.1 (CSNK1A1P, ENST00000430593)**

**
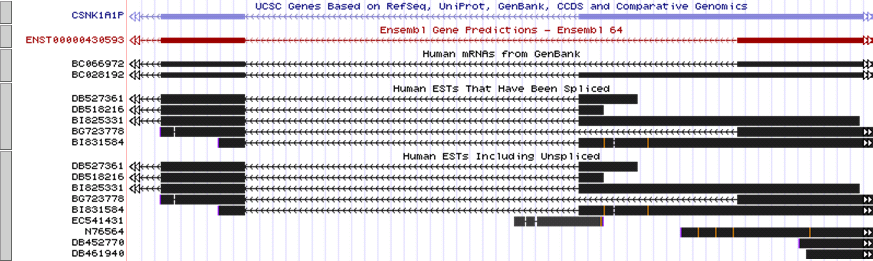
**
