# Supplementary material for: Newly evolved introns in human retrogenes provide novel insights into their evolutionary roles
Source: BMC Evol Biol. 2012 Jul 28;12:128. doi: 10.1186/1471-2148-12-128 (PMC3565874; doi:10.1186/1471-2148-12-128)
Supplement: Additional file 3 — List of human tissues sampled for the experiments. This file lists the human tissues that we used for the experiments to validate the existence of retrogene introns [file 1471-2148-12-128-S3.doc]

**Additional file 3**

**List of human tissues sampled for the experiments.**

| Serial No. | Tissue | Serial No. | Tissue |
| --- | --- | --- | --- |
| 1 | colon | 9 | adrenal gland |
| 2 | umbilical cord | 10 | uterus |
| 3 | saphenous vein | 11 | skeletal muscle |
| 4 | gallbladder | 12 | kidney |
| 5 | foreskin | 13 | thyroid gland |
| 6 | lung | 14 | stomach |
| 7 | oesophagus | 15 | liver |
| 8 | tonsil | 16 | bladder |
