# Supplementary material for: Newly evolved introns in human retrogenes provide novel insights into their evolutionary roles
Source: BMC Evol Biol. 2012 Jul 28;12:128. doi: 10.1186/1471-2148-12-128 (PMC3565874; doi:10.1186/1471-2148-12-128)
Supplement: Additional file 4 — Experimental validation of retrogene introns in TMEM14D and HSP90AA4P. This file shows the experimental results for validating the existence of retrogene introns [file 1471-2148-12-128-S4.doc]

**Additional file 4**

**Experimental validation of retrogene introns in TMEM14D and HSP90AA4P.**

For each case, the upper figure is the RT-PCR results (A, different tissues were numbered according to Additional File 2, while the upper band is corresponding to the mRNA from the parent and the lower one from the retrogene; B, DNA fragments of the housekeeping gene glyceraldehyde-3-phosphate dehydrogenase (omitted in HSP90AA4P)). The lower figure is the alignment (program, Water of Emboss [S1]) of sequencing results. ‘Sbjct’ is the band in smaller size and ‘Query’ is the bigger one. Bases covered by grayish box marked the beginnings and endings of the intronic region validated by experiments and they both are in accordance with the annotations from UCSC [S2, S3].

**References**

S1. Rice P, Longden I, Bleasby A: **EMBOSS: The European Molecular Biology Open Software Suite**. *Trends Gene* 2000, **16(6)**:276-277.

S2. Karolchik D, Hinrichs AS, Furey TS, Roskin KM, Sugnet CW, Haussler D, Kent WJ: **The UCSC Table Browser data retrieval tool.** *Nucleic Acids Res* 2004, **32(Database issue)**:D493-496.

S3. Kuhn RM, Karolchik D, Zweig AS, Wang T, Smith KE, Rosenbloom KR, Rhead B, Raney BJ, Pohl A, Pheasant M, Meyer L, Hsu F, Hinrichs AS, Harte RA, Giardine B, Fujita P, Diekhans M, Dreszer T, Clawson H, Barber GP, Haussler D, Kent WJ: **The UCSC Genome Browser Database: update 2009**. *Nucleic Acids Res* 2009, **37(Database issue)**:D755-761.

**TMEM14D**


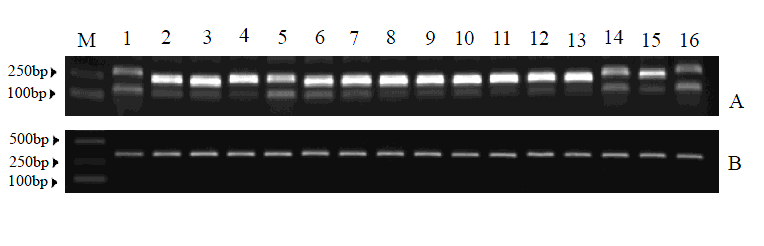


Query TCTTCCCATTAGTGCCTTTGCATTGGTTTGGCTTTGGCTACACAGCACTG

||||||||||||||||||||||||||||||||||||||||||||||||||

Sbjct TCTTCCCATTAGTGCCTTTGCATTGGTTTGGCTTTGGCTACACAGCACTG

Query GTTGTTTCTGGTGGGATCGTTGGCTATGTAAAAACAGGCAGCGTGCCGTC |||||||||||||||||||||||||||||||||||||

Sbjct GTTGTTTCTGGTGGGATCGTTGGCTATGTAAAAACAG-------------

Query CCTGGCTGCAGGGCTGCTCTTCGGCAGTCTAGCCGGCCTGGGTGCTTACC

Sbjct --------------------------------------------------

Query AGCTGTATCAGGATCCAAGGAACGTTTGGGGTTTCCTAGCCGCTACATCT

_____|||||||||||

Sbjct ---------------------------------------CCGCTACATCT

Query GTTACTTTTGTTGGTATTATGGGAATGAGA

|||||||||||||||.||||||||||||||

Sbjct GTTACTTTTGTTGGTGTTATGGGAATGAGA

**HSP90AA4P**


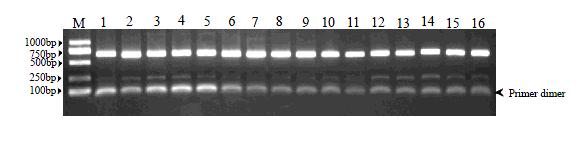


Query GACCAATGACTGGGAAGATCACTTGGCAGTGAAGCATTTTTCAGTTGAAG

|||||.|.|||||||||||.||||||||||||||||||||||||||||||

Sbjct GACCATTAACTGGGAAGATTACTTGGCAGTGAAGCATTTTTCAGTTGAAG

Query GACAGTTGGAATTCAGAGCCCTTCTATTTGTCCCACGACGTGCTCCTTTT

||||||||||||||||||||.||||||||||||||||||.||||||||||

Sbjct GACAGTTGGAATTCAGAGCCTTTCTATTTGTCCCACGACTTGCTCCTTTT

Query GATCTGTTTGAAAACAGAAAGAAAAAGAACAACATCAAATTGTATGTACG

||.|||||.||||.||||||||||||||||||.||||||||||.||.|||

Sbjct GAGCTGTTGGAAACCAGAAAGAAAAAGAACAAAATCAAATTGTCTGCACG

Query CAGAGTTTTCATCATGGATAACTGTGAGGAGCTAATCCCTGAATATCTGA

|||||.|.||||||||||||||||||||||||||||||||||||||||||

Sbjct CAGAGATCTCATCATGGATAACTGTGAGGAGCTAATCCCTGAATATCTGA

Query ACTTCATTAGAGGGGTGGTAGACTCGGAGGATCTCCCTCTAAACATATCC

|||||||.|||||||||||||||||||||||||||||||||||.||.|.|

Sbjct ACTTCATCAGAGGGGTGGTAGACTCGGAGGATCTCCCTCTAAATATTTTC

Query CGTGAGATGTTGCAACAAAGCAAAATTTTGAAAGTTATCAGGAAGAATTT

|

Sbjct C-------------------------------------------------

Query GGTCAAAAAATGCTTAGAACTCTTTACTGAACTGGCGGAAGATAAAGAGA

Sbjct --------------------------------------------------

Query ACTACAAGAAATTCTATGAGCAGTTCTCTAAAAACATAAAGCTTGGAATA

Sbjct --------------------------------------------------

Query CACGAAGACTCTCAAAATCGGAAGAAGCTTTCAGAGCTGTTAAGGTACTA

Sbjct --------------------------------------------------

Query CACATCTGCCTCTGGTGATGAGATGGTTTCTCTCAAGGACTACTGCACCA

Sbjct --------------------------------------------------

Query GAATGAAGGAGAACCAGAAACATATCTATTATATCACAGGTGAGACCAAG

___________________________________________________|||||||||||

Sbjct ---------------------------------------GTGAGACCAAG

Query GACCAGGTAGCTAACTCAGCCTTTGTGGAACGTCTTCGGAAACATGGCTT

||||||||||||||||||.||.|||||.||||||||.|||||||||||||

Sbjct GACCAGGTAGCTAACTCAACCATTGTGCAACGTCTTTGGAAACATGGCTT

Query AGAAGTGATCTATATGATTGAGCCCATTGATGA

.|||||||||||||.|||.||||||||||||||

Sbjct GGAAGTGATCTATACGATCGAGCCCATTGATGA
