# Supplementary material for: Newly evolved introns in human retrogenes provide novel insights into their evolutionary roles
Source: BMC Evol Biol. 2012 Jul 28;12:128. doi: 10.1186/1471-2148-12-128 (PMC3565874; doi:10.1186/1471-2148-12-128)

**Additional file 5**

**Phylogenetic tree for vertebrates.**

The timeline and the divergence times of species are constructed basing on UCSC genome browser database [S1, S2], as well as other sources [S3-S7]. Different evolution branches are labeled from 0 to 12.

**References**

S1. Karolchik D, Hinrichs AS, Furey TS, Roskin KM, Sugnet CW, Haussler D, Kent WJ: **The UCSC Table Browser data retrieval tool.** *Nucleic Acids Res* 2004, **32(Database issue)**:D493-496.

S2. Kuhn RM, Karolchik D, Zweig AS, Wang T, Smith KE, Rosenbloom KR, Rhead B, Raney BJ, Pohl A, Pheasant M, Meyer L, Hsu F, Hinrichs AS, Harte RA, Giardine B, Fujita P, Diekhans M, Dreszer T, Clawson H, Barber GP, Haussler D, Kent WJ: **The UCSC Genome Browser Database: update 2009**. *Nucleic Acids Res* 2009, **37(Database issue)**:D755-761.

S3. Thomas JW, Touchman JW: **Vertebrate genome sequencing: building a backbone for comparative genomics**. *Trends Genet* 2002, **18(2)**:104-108.

S4. Zhao S, Shetty J, Hou L, Delcher A, Zhu B, Osoegawa K, de Jong P, Nierman WC, Strausberg RL, Fraser CM: **Human, mouse, and rat genome large-scale rearrangements: stability versus speciation**. *Genome* *Res* 2004, **14(10A)**:1851-1860.

S5. Falkowski PG, Katz ME, Milligan AJ, Fennel K, Cramer BS, Aubry MP, Berner RA, Novacek MJ, Zapol WM: **The rise of oxygen over the past 205 million years and the evolution of large placental** **mammals**. *Science* 2005, **309(5744)**:2202-2204.

S6. Waters PD, Delbridge ML, Deakin JE, El-Mogharbel N, Kirby PJ, Carvalho-Silva DR, Graves JA: **Autosomal location of genes from the conserved mammalian X in the platypus (*Ornithorhynchus*** ***anatinus*): implications for mammalian sex chromosome evolution**. *Chromosome Res* 2005, **13(4)**:401-410.

S7. Zhang YE, Vibranovski MD, Landback P, Marais GA, Long M: **Chromosomal redistribution of male-biased genes in mammalian evolution with two bursts of gene gain on the X chromosome**. *PLoS Biol* 2010, **8(10)**:e1000494.


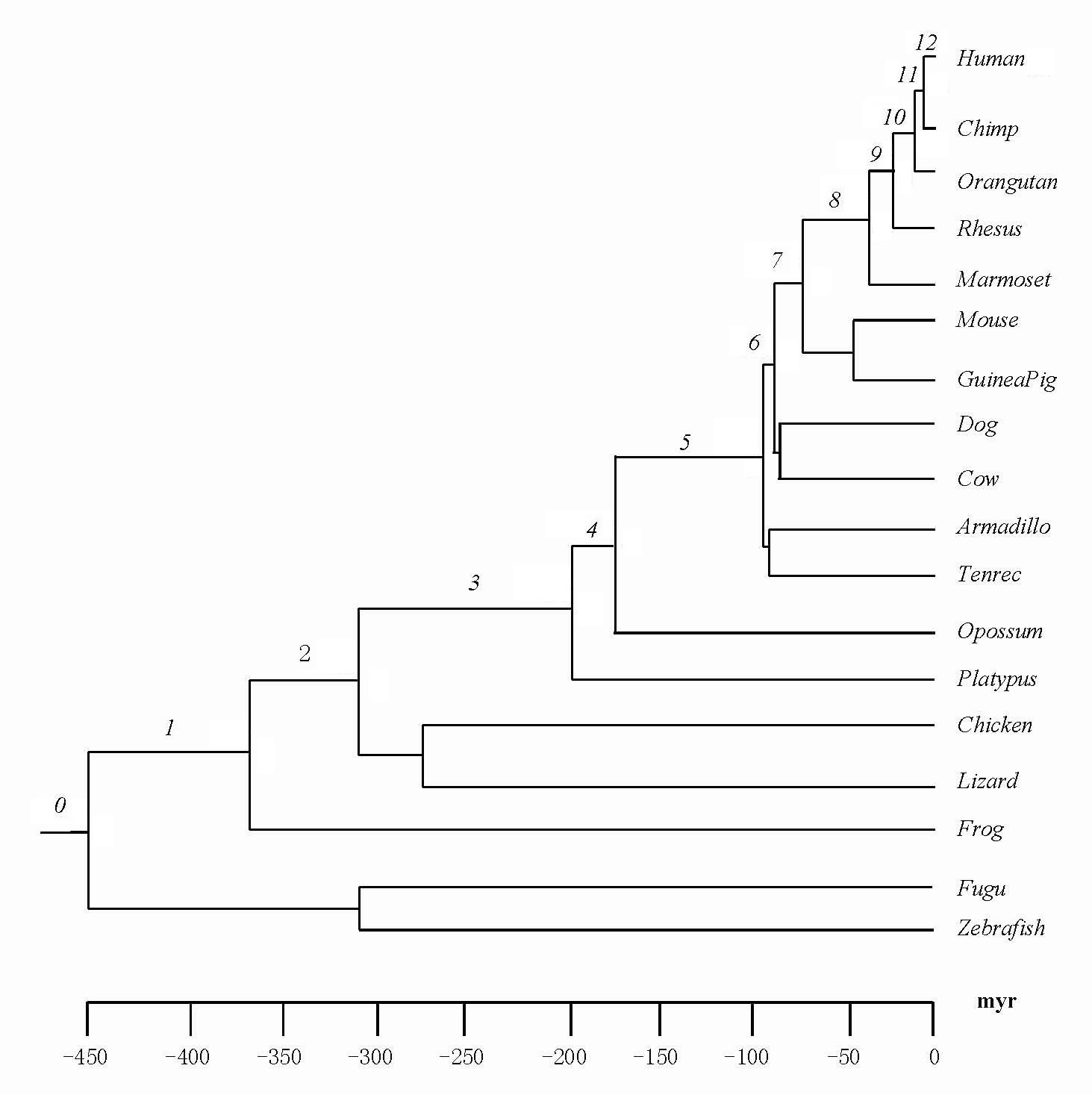

Supplement: Additional file 5 — Phylogenetic tree for vertebrates. A diagram of the phylogenetic tree for vertebrates [file 1471-2148-12-128-S5.doc]
