# Supplementary material for: Newly evolved introns in human retrogenes provide novel insights into their evolutionary roles
Source: BMC Evol Biol. 2012 Jul 28;12:128. doi: 10.1186/1471-2148-12-128 (PMC3565874; doi:10.1186/1471-2148-12-128)
Supplement: Additional file 6 — Chromosome and time of origin of intronized retrogenes. This file shows the origination times of intronized retrogenes [file 1471-2148-12-128-S6.doc]

**Additional file 6**

**Chromosome and time of origin of intronized retrogenes.**

| Gene Symbol | Chr | Parent Chr | Branch |
| --- | --- | --- | --- |
| TMEM14D | 10 | 6 | 12 |
| HSP90B2P | 15 | 12 | 9 |
| HSP90AA4P | 4 | 14 | 9 |
| HSP90AA5P | 3 | 14 | 8 |
| AC019016.1 | 15 | 5 | 0 |

Column ‘Branch’ follows the labeling of branches in Additional file 5.

.
