# Supplementary material for: Newly evolved introns in human retrogenes provide novel insights into their evolutionary roles
Source: BMC Evol Biol. 2012 Jul 28;12:128. doi: 10.1186/1471-2148-12-128 (PMC3565874; doi:10.1186/1471-2148-12-128)
Supplement: Additional file 7 — Chromosome and time of origin of retrogenes that gained introns by chimerization. This file shows the origination times of retrogenes that gained introns by chimerization [file 1471-2148-12-128-S7.doc]

**Additional file 7**

**Chromosome and time of origin of retrogenes that gained introns by chimerization [S1]**.

| Gene Symbol | Chr | Parent Chr | Branch |
| --- | --- | --- | --- |
| DUSP5P | 1 | 10 | 12 |
| PLEKHA9 | 12 | 7 | 10 |
| RBMXL1 | 1 | X | 9 |
| FUNDC2P2 | 2 | X | 9 |
| SPIN2B | X | 9 | 8 |
| UTP14C | 13 | X | 6 |
| FAM50B | 6 | X | 5 |
| TAF9 | 5 | X | 5 |
| HNRPF | 10 | 5 | 5 |
| HNRPH2 | X | 5 | 5 |
| HMGN4 | 6 | 1 | 5 |
| NXT1 | 20 | X | 5 |
| COX7B2 | 4 | X | 5 |
| ATP6V1E2 | 2 | 22 | 5 |
| RPL36AL | 14 | X | 4 |
| FAM113B | 12 | 20 | 4 |
| ARD1B | 4 | X | 4 |
| NUP62 | 19 | X | 2 |
| SLC35A4 | 5 | 1 | 1 |
| RHOG | 11 | 7 | 0 |
| ALDH1B1 | 9 | 12 | 0 |
| RHOH | 4 | 7 | 0 |
| HSPA1L | 6 | 11 | 0 |
| DNJB8 | 3 | 7 | 0 |
| HSPA2 | 14 | 11 | 0 |
| ARF6 | 14 | 12 | 0 |
| SOD3 | 4 | 21 | 0 |

Column ‘Branch’ follows the labeling of branchs in Additional file 5.

**References**

S1. Fablet M, Bueno M, Potrzebowski L, Kaessmann H: **Evolutionary origin and functions of retrogene introns**. *Mol Biol Evol* 2009, **26(9)**:2147-2156.
