# Supplementary material for: Newly evolved introns in human retrogenes provide novel insights into their evolutionary roles
Source: BMC Evol Biol. 2012 Jul 28;12:128. doi: 10.1186/1471-2148-12-128 (PMC3565874; doi:10.1186/1471-2148-12-128)
Supplement: Additional file 8 — Transcription annotations (from the UCSC Genome Browser database) of retrogene introns in the parental gene. This file contains snapshots from the UCSC Genome Browser database displaying transcription annotations of retrogene introns in the parental gene [file 1471-2148-12-128-S8.doc]

**Additional file 8**

**Transcription annotations (from the UCSC Genome Browser database [S1,S2]) of retrogene introns in the parental gene.**

Above each snapshot is an intron symbol, in a format, gene name plus a serial number following the hyphen. For example, ‘HSP90B2P-1’ indicates the first intron (in the direction from 5′ to 3′) in HSP90B2P. If a retrogene evolved only one intron (TMEM14D), the intron is represented by the gene name. For the two alternatively spliced introns of AC019016.1, ‘-L’ indicates the larger one, while ‘-S’ is the smaller one. The red band below the text “Your Sequence from Blat Search” is the corresponding region of the retrogene intron in the parental gene.

**References**

S1. Karolchik D, Hinrichs AS, Furey TS, Roskin KM, Sugnet CW, Haussler D, Kent WJ: **The UCSC Table Browser data retrieval tool.** *Nucleic Acids Res* 2004, **32(Database issue)**:D493-496.

S2. Kuhn RM, Karolchik D, Zweig AS, Wang T, Smith KE, Rosenbloom KR, Rhead B, Raney BJ, Pohl A, Pheasant M, Meyer L, Hsu F, Hinrichs AS, Harte RA, Giardine B, Fujita P, Diekhans M, Dreszer T, Clawson H, Barber GP, Haussler D, Kent WJ: **The UCSC Genome Browser Database: update 2009**. *Nucleic Acids Res* 2009, **37(Database issue)**:D755-761.

**TMEM14D**

**
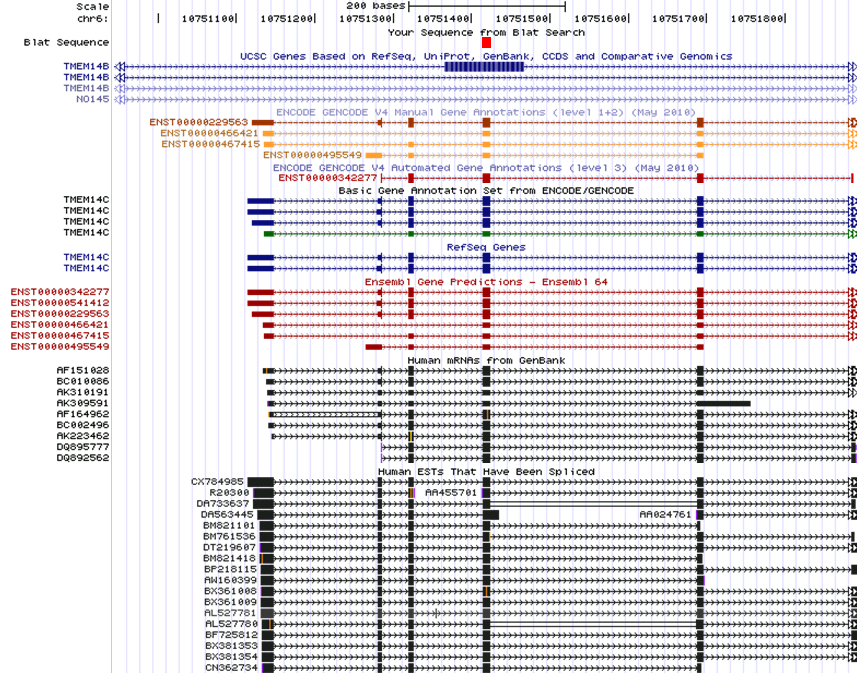
**

**HSP90B2P-1**

**
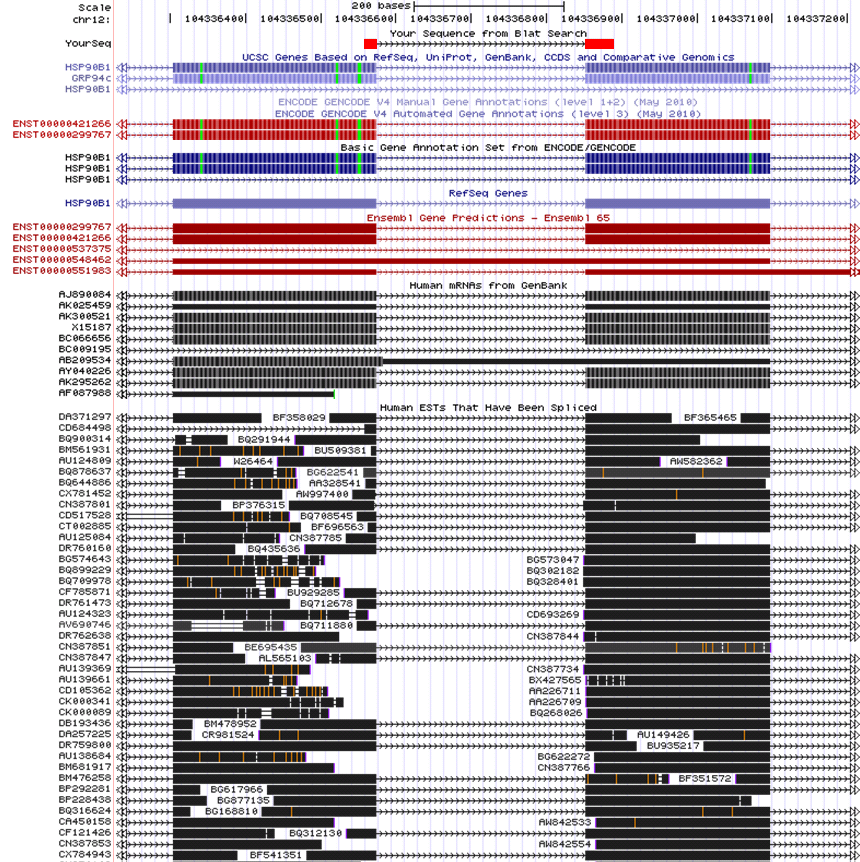
**

**HSP90B2P-2**

**
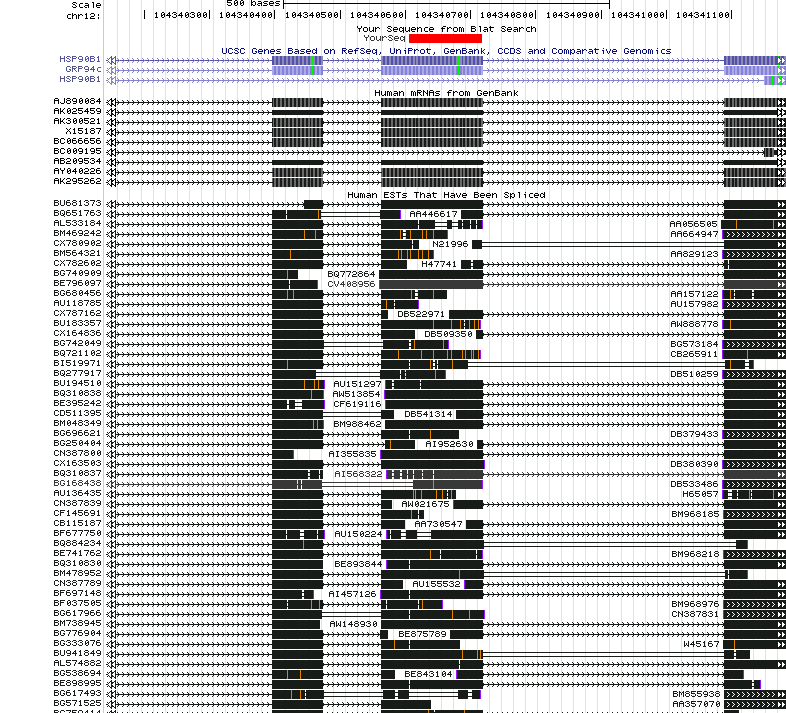
**

**HSP90AA4P-1**

**
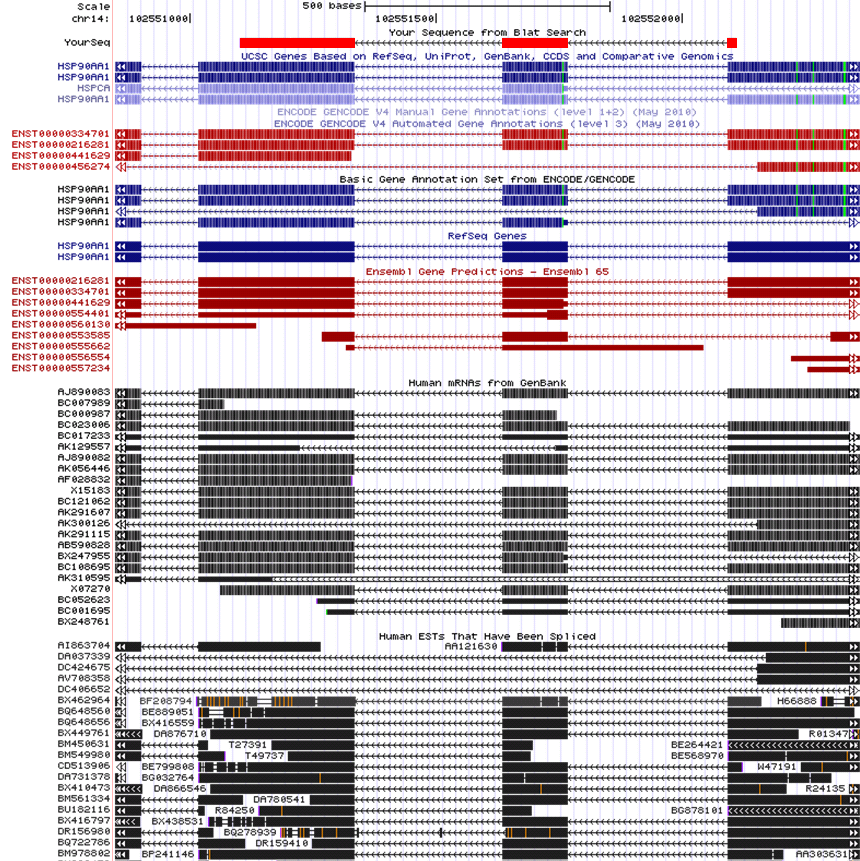
**

**HSP90AA4P-2**

**
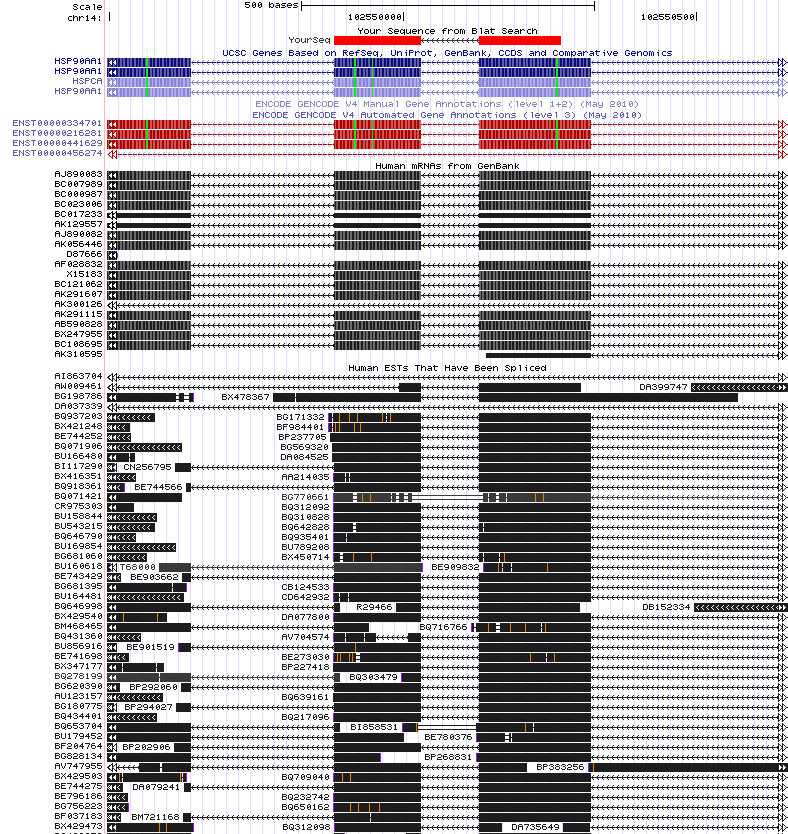
**

**HSP90AA4P-3**

**
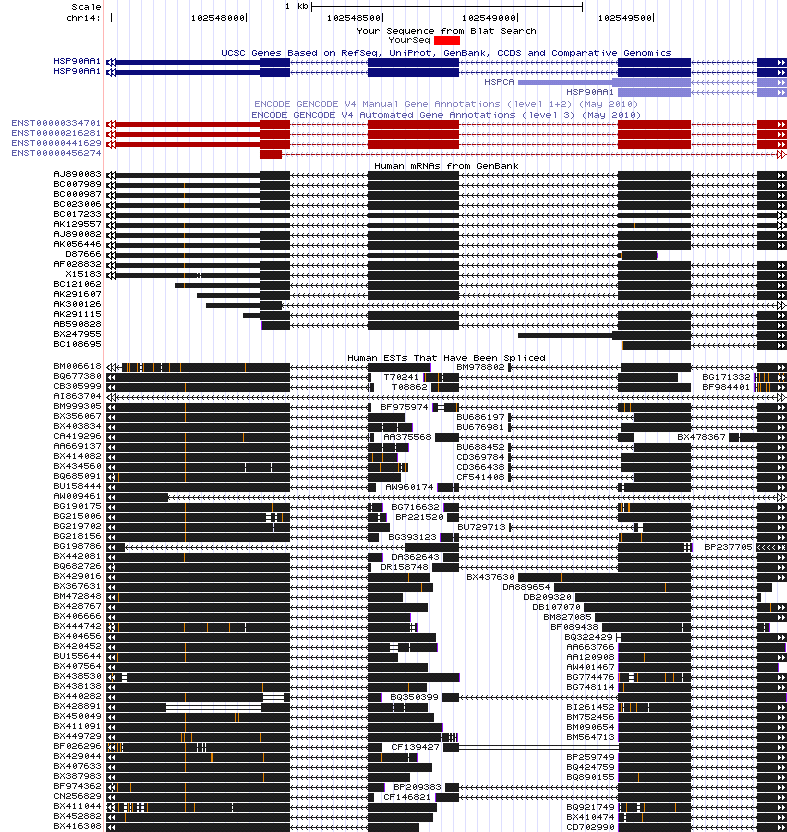
**

**HSP90AA5P-1**

**
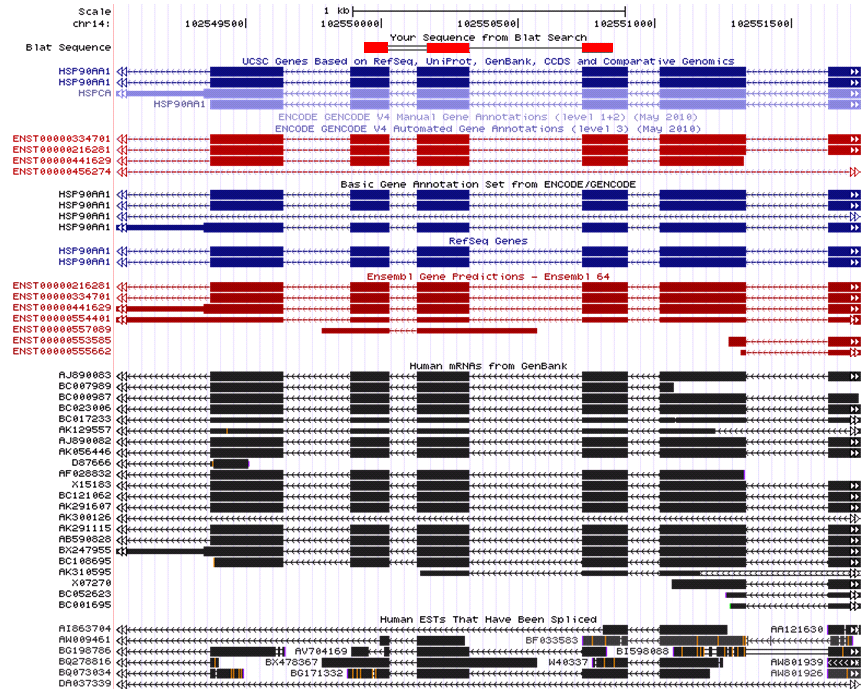
**

**HSP90AA5P-2**

**
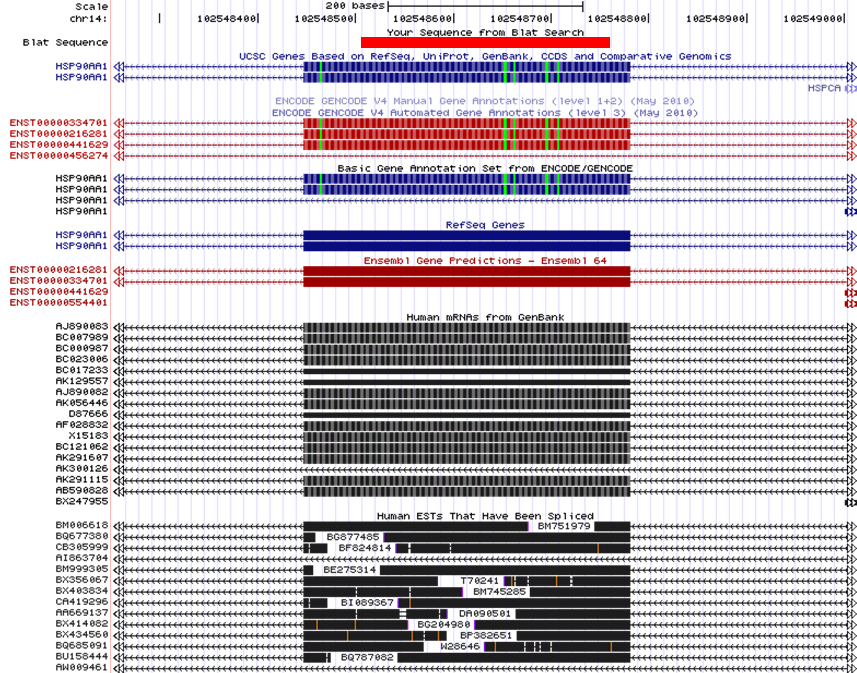
**

**AC019016.1-L**

**
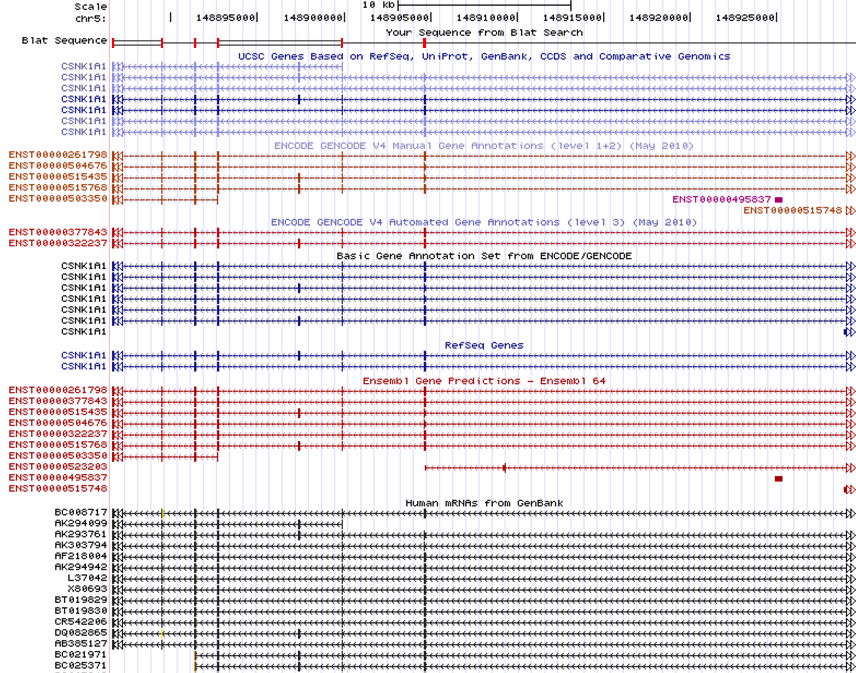
**

**AC019016.1-S**

**
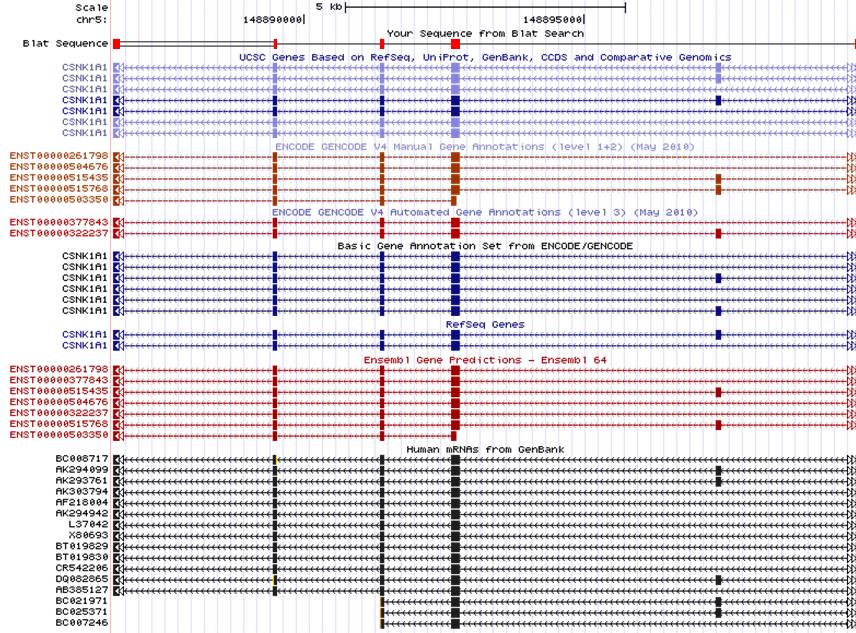
**
