# Supplementary material for: Newly evolved introns in human retrogenes provide novel insights into their evolutionary roles
Source: BMC Evol Biol. 2012 Jul 28;12:128. doi: 10.1186/1471-2148-12-128 (PMC3565874; doi:10.1186/1471-2148-12-128)
Supplement: Additional file 9 — Sequences of primer pairs used to amplify the retrogenes and their parents. A table that lists primer pairs we used to amplify the retrogenes and their parents [file 1471-2148-12-128-S9.doc]

**Additional file 9**

**Sequences of primer pairs used to amplify the retrogenes and their parents.**

| Retro-parental pair | | Sense primer | Anti-sense primer |
| --- | --- | --- | --- |
| TMEM14D,  TMEM14B | CTTCCCATTAGTGCCTTTG | | AGGATCTCATTCCCATAACAC |
| HSP90AA4P,  HSP90AA1 | GACCAATGACTGGGAAGATC | | TCATCAATGGGCTCAATCA |
