# Supplementary material for: Newly evolved introns in human retrogenes provide novel insights into their evolutionary roles
Source: BMC Evol Biol. 2012 Jul 28;12:128. doi: 10.1186/1471-2148-12-128 (PMC3565874; doi:10.1186/1471-2148-12-128)
Supplement: Additional file 10 — Protein-level alignments of intron-gain retrogenes (“Sbjct”) and their parents (“Query”) by GeneWise. This file contains alignments of intron-gain retrogenes and their parents in protein level [file 1471-2148-12-128-S10.pdf]

Additional file 10

Protein-level alignments of intron gain retrogenes (“Sbjct”) and their parents (“Query”) by GeneWise [S1].

Each alignment shows the protein sequence of the parental gene on the first line, followed by a line indicating the similarity level of the match followed by a line representing the translation of the DNA sequence of the retrogene. If the intervening sequence of the retrogene is long, it is indicated in the square brackets. If the intervening sequence of the retrogene is short and not divisible by 3, it is indicated by '!'. (for specifics, see the description of the output format of GeneWise (http://www.ebi.ac.uk/Tools/Wise2/)). Red downward arrows indicate the exon-exon borders of parental genes and the blue upward arrows and connecting lines mark the new intron in the retrogene. In XXyac-R12DG2.2, the retrogene is also annotated to be embedded near the 3’ end of an ncRNA gene candidate (LOC100190939, Additional file 12). In CSMD3, the green upward arrows and connecting lines mark the intragenic region in the retroposed sequence. The retrogene is an alternative translation start point (Additional file 12). In WBP2NL, the retrogene is located in its first intron, but could be transcribed at sometimes (Additional file 1 and 2). Besides, the intronic region at XXyac-R12DG2.2 and AC019016.1 could be spliced in two patterns.

References

S1. Birney E, Clamp M, Durbin R: **GeneWise and Genomewise**. *Genome Res* 2004, **14(5)**:988-995.

TMEM14D

```
Query     1  MEKPLFPLVPLHWFGFGYTALVVSGGIVGYVKTGSVPSLAAGLLFGSLA
             MEKPLFPLVPLHWFGFGYTALVVSGGIVGYVKTG   PSLAAGLLFGSLA
Sbjct     2  MEKPLFPLVPLHWFGFGYTALVVSGGIVGYVKTGRAPSLAAGLLFGSLA

Query    50  GLGAYQLYQDPRNVWGFLAATSVTFVGVMGMRSYYYGKFMPVGLIAGAS
             G+GAYQLYQDPRNVW FLAATSVTFVG+MGMRSYYYGKFMPVGLIAGAS
Sbjct   149  GVGAYQLYQDPRNVWDFLAATSVTFVGIMGMRSYYYGKFMPVGLIAGAS

Query    99  LLMAAKVGVRMLMTSD
             LLMAAKVGVRMLMTSD
Sbjct   296  LLMAAKVGVRMLMTSD
```

RPS3AP5

```
Query     1  MAVGKNKRLTKGGKKGAKKKVVDPFSKKDWYDVKAPAMFNIRNIGKTLV
             M V KNKRLTKGGKKGAKKKVVDPFSKKDWYDV APAMFNIRNIGKTLV
Sbjct     2  MVVDKNKRLTKGGKKGAKKKVVDPFSKKDWYDVNAPAMFNIRNIGKTLV

Query    50  TRTQGTKIASDGLKGRVFEVSLADLQNDEVAFRKFKLITEDVQGKNCLT
             TRTQGTKIASDG   RVFEVSLADLQNDEVAFRKFKLITEDVQGKNCLT
Sbjct   149  TRTQGTKIASDG---RVFEVSLADLQNDEVAFRKFKLITEDVQGKNCLT

Query    99  NFHGMDLTRDKMCSMVKKWQTMIEAHVDVKTTDGYLLRLFCVGFTKKRN
             NFHG+DLT DKMCSMVKKWQTMIEAHVDVKTTDGYLLRLFCVGFTKKRN
Sbjct   287  NFHGVDLTSDKMCSMVKKWQTMIEAHVDVKTTDGYLLRLFCVGFTKKRN

Query   148  NQIRKTSYAQHQQVR-----------QIRKKMMEIMTREVQTNDLKEVV
             NQIRKTSYAQHQQV            QIRKKMMEIMTREVQTNDLKEVV
Sbjct   434  NQIRKTSYAQHQQV!KSGRPLMLSTSQIRKKMMEIMTREVQTNDLKEVV

Query   186  NKLIPDSIGKDIEKACQSIYPLHDVFVRKVKMLKKPKFELGKLMELHGE
             NKLIPDSIGKD+EKACQSIYPLHDVFVRKVKMLKKPKFELGKLMELHGE
Sbjct   583  NKLIPDSIGKDVEKACQSIYPLHDVFVRKVKMLKKPKFELGKLMELHGE

Query   235  GSSSGKATGDETGAKVERADGYEPPVQESV
             G SSGKATGDETG KVERADGYE PVQESV
Sbjct   730  GCSSGKATGDETGVKVERADGYELPVQESV
```

XXyac-R12DG2.2

```
Query     1  MARGGRGRRLGLALGLLLALVLAPRVLRAKPTVRKERVVRPDSELGERP
             MARGGR   LGLALGLLLALVLAPRVLRAKPTVRKERVVRPDSELGERP
Sbjct     2  MARGGR---LGLALGLLLALVLAPRVLRAKPTVRKERVVRPDSELGERP
```

```
Query     50  PEDNQSFQYDHEAFLGKEDSKTFDQLTPDESKERLGKIVDRIDNDGDGF
              PEDNQSFQYDHEAFLGKEDSK FD+LTPDESKERLGKIVDRIDNDG GF
Sbjct    140  PEDNQSFQYDHEAFLGKEDSKNFDELTPDESKERLGKIVDRIDNDGYGF

Query     99  VTTEELKTWIKRVQKRYIFDNVAKVWKDYDRDKDDKISWEEYKQATYGY
              VTTEELKTWIKRVQKRYIFDNVAKVWKDYDRDKDD ISWEEYKQATYGY
Sbjct    287  VTTEELKTWIKRVQKRYIFDNVAKVWKDYDRDKDDNISWEEYKQATYGY

Query    148  YLGNPAEFHDSSDHHTFKKMLPRDERRFKAADLNGDLTATREEFTAFLH
              Y GNPAEFHDSSDHHTFKKMLPRDERRFKAADLNGDLTAT EEFTAFLH
Sbjct    434  Y!GNPAEFHDSSDHHTFKKMLPRDERRFKAADLNGDLTATPEEFTAFLH

Query    197  PEEFEHMKEIVVLETLEDIDKNGDGFVDQDEYIADMFSHEENGPEPDWV
              PEEFEHMKEIV LETLEDIDKNGDGFVDQDEYIADMFSHEENGPEPDWV
Sbjct    583  PEEFEHMKEIVALETLEDIDKNGDGFVDQDEYIADMFSHEENGPEPDWV

Query    246  LSEREQFNEFRDLNKDGKLDKDEIRHWILPQDYDHAQAEARHLVYESDK
              LSEREQFNEFRDLNKDGKLDKDEIRHWILPQDYDHAQAEARHLVYESDK
Sbjct    730  LSEREQFNEFRDLNKDGKLDKDEIRHWILPQDYDHAQAEARHLVYESDK

Query    295  NKDEKLTKEEILENWNMFVGSQATNYGEDLTKNHDEL
              NKDEKLTKEEILENWNMFVGSQATNYGEDLTKNHDEL
Sbjct    877  NKDEKLTKEEILENWNMFVGSQATNYGEDLTKNHDEL
```

HSP90B2P

```
Query      8  GLCCVLLTFGSVRADDEVDVDGTVEEDLGKSREGSRTDDEVVQREEEAI
              GLCCVLLTFGSVRA DEVDV+GTVEEDLGKSR+GSR DDEVVQREEEAI
Sbjct     24  GLCCVLLTFGSVRAVDEVDVEGTVEEDLGKSRQGSRMDDEVVQREEEAI

Query     57  QLDGLNASQIRELREKSEKFAFQAEVNRMMKLIINSLYKNKEIFLRELI
              QLD LNASQIRELREK EKFAFQAEVNRMMKLIINSLY+NKEIFLRELI
Sbjct    171  QLDKLNASQIRELREKLEKFAFQAEVNRMMKLIINSLYENKEIFLRELI

Query    106  SNASDALDKIRLISLTDENALSGNEELTVKIKCDKEKNLLHVTDTGVGM
                  DAL KIRLISLT+ENALSGNEELTVKIKCDK KNLLHVTDTGVGM
Sbjct    318  !---DALVKIRLISLTNENALSGNEELTVKIKCDKVKNLLHVTDTGVGM

Query    155  TREELVKNLGTIAKSGTSEFLNKMTEAQEDGQSTSELIGQFGVGFYSAF
              TREELVKNLGTIAKS T+EFLNKMTEAQEDGQSTSELIGQFGV FYS F
Sbjct    455  TREELVKNLGTIAKSXTNEFLNKMTEAQEDGQSTSELIGQFGVSFYSIF

Query    204  LVADKVIVTSKHNNDTQHIWESDSNEFSVIADPRGNTLGRGTTITLVLK
              LVADKVIVTSKHNNDTQ+IWES+SNE+SVIADPRGNTLGRGTTITLVLK
Sbjct    602  LVADKVIVTSKHNNDTQYIWESESNEYSVIADPRGNTLGRGTTITLVLK
```

```
Query    253  EEASDYLELDTIKNLVKKYSQFINFPIYVWSSKTETVEEPMEEEEAAKE
              E+A DYL LDTIKNL+ KYSQFINFPIYVWSSKTETV+EP +EE AAKE
Sbjct    749  EKAFDYLALDTIKNLI!KYSQFINFPIYVWSSKTETVKEP!KEEAAAKE

Query    302  EKEESDDEAAVEEEEEEKKPKTKKVEKTVWDWELMNDIKPIWQRPSKEV
              EKEES DEAAVEEEEE++KPKTKKVEKTV +WELMNDIKPIWQRPS EV
Sbjct    894  EKEESYDEAAVEEEEEKQKPKTKKVEKTVCEWELMNDIKPIWQRPS!EV

Query    351  EEDEYKAFYKSFSKESDDPMAYIHFTAEGEVTFKSILFVPTSAPRGLFD
              E DEYKAF KSFSKESDDP+A IHFTAEGEVTFKSILFVPT  PRGLFD
Sbjct   1040  E-DEYKAFCKSFSKESDDPVACIHFTAEGEVTFKSILFVPTFVPRGLFD

Query    400  EYGSKKSDYIKLYVRRVFITDDFHDMMPKYLNFVKGVVDSDDLPLNVSR
              EYGSKKSDYIKLYVR VFITDDF D MPK LNFVKGVVDS  L LNVS
Sbjct   1184  EYGSKKSDYIKLYVRCVFITDDFRDTMPKNLNFVKGVVDSGGLSLNVSC

Query    449  ETLQQHKLLKVIRKKLVRKTLDMIKKIADDKYNDTFWKEFGTNIKLGVI
              ETLQQHKLLKVIRKKLV KTLDMIKKIAD+KYNDTFWKEFGTNIKLGVI
Sbjct   1331  ETLQQHKLLKVIRKKLVHKTLDMIKKIADEKYNDTFWKEFGTNIKLGVI

Query    498  EDHSNRTRLAKLLRFQSSHHPTDITSLDQYVERMKEKQDKIYFMAGSSR
              EDHSNRT LAKLLRFQSSHHP DITSL Q VERMKEKQDKI +MAGSSR
Sbjct   1478  EDHSNRTCLAKLLRFQSSHHPADITSLHQDVERMKEKQDKICLMAGSSR

Query    547  KEAESSPFVERLLKKGYEVIYLTEPVDEYCIQALPEFDGKRFQNVAKEG
              KEAESSPFV RLLK+GYEVIYLTEPV EYCIQALPEFDGKRFQNVAKEG
Sbjct   1625  KEAESSPFVXRLLKRGYEVIYLTEPVVEYCIQALPEFDGKRFQNVAKEG

Query    596  VKFDESEKTKESREAVEKEFEPLLNWMKDKALKDKIEKAVVSQRLTESP
              VKFD+SEKTKES EAVEKEFEPL NW+KDKA+KDKIEKA+VSQ LTES
Sbjct   1772  VKFDDSEKTKESHEAVEKEFEPLPNWVKDKAIKDKIEKAMVSQCLTESL

Query    645  CALVASQYGWSGNMERIMKAQAYQTGKDISTNYYASQKKTFEINPRHPL
              CALVASQYGWSGNMERIMKAQAYQTGK ISTNY+AS+KKTFEINPRHPL
Sbjct   1919  CALVASQYGWSGNMERIMKAQAYQTGKGISTNYHASRKKTFEINPRHPL

Query    694  IRDMLRRIKEDEDDKTVLDLAVVLFETAT--LRSGYLLPDTKAYGDRIE
              IRDMLRRIKEDEDDKTVLDLAVVLFETA   +RSG LLPDTKAYGDRIE
Sbjct   2066  IRDMLRRIKEDEDDKTVLDLAVVLFETA!AVIRSGCLLPDTKAYGDRIE

Query    741  RMLRLSLNIDPDAKVEEEPEEEPEETAEDTTEDTEQDEDEEMDVGTDEE
              RM  LSLN  PDAKVEE P+EEPEETAED     EQD+D+EMDVGTDEE
Sbjct   2215  RMPCLSLNT!PDAKVEE-PDEEPEETAEDK----EQDKDKEMDVGTDEE

Query    790  -EETAKESTAEKDEL
                ETAKESTAEKDEL
Sbjct   2345  KQETAKESTAEKDEL
```

HSP90AA4P

```
Query    1    MPEETQTQDQPMEEEEVETFAFQAEIAQLMSLIINTFYSNKEIFLRELI
              MPEETQTQDQP EE EVETF FQAEIAQLMSL+INTFY NKEIFLRELI
Sbjct    6    MPEETQTQDQPTEEXEVETFTFQAEIAQLMSLFINTFYLNKEIFLRELI

Query   50    SNSSDALDKIRYESLTDPSKLDSGKELHINLIPNKQDRTLTIVDTGIGM
              SN SDALDKI  ESLTDPSKLDSGKE HI+LIPNKQDRTLTIVDTGIGM
Sbjct  153    SNLSDALDKILXESLTDPSKLDSGKEPHISLIPNKQDRTLTIVDTGIGM

Query   99    TKADLINNLGTIAKSGTKAFMEALQAGADISMIGQFGVGFYSAYLVAEK
              TKADLINNLGTI KS TK FME LQAGADISMIGQF VGFYSAY VAEK
Sbjct  300    TKADLINNLGTITKSETKVFMEVLQAGADISMIGQFSVGFYSAYSVAEK

Query  148    VTVITKHNDDEQYAWESSAGGSFTVRTDTGEPMGRGTKVILHLKEDQTE
              VTVITKHN+DEQYAWESS  GSFTVRTDTGEP+G GTKVIL LKEDQTE
Sbjct  447    VTVITKHNNDEQYAWESSLRGSFTVRTDTGEPIGHGTKVILPLKEDQTE

Query  197    YLEERRIKEIVKKHSQFIGYPITLFVEKERDKEVSDDEAEEKEDKEEEK
              YLEER+I EIVKKHS FIGYP+TLFVEK+ DKEVS+DEAEEKEDKEEEK
Sbjct  594    YLEERKINEIVKKHS!FIGYPVTLFVEKKHDKEVSNDEAEEKEDKEEEK

Query  246    EKEEKESEDKPEIEDVGSDEEEE-KKDGD----KKKKKKIKEKYIDQEE
              EKEEKESEDKPEIEDVGSDEEEE KKDGD    KKKKKKIKEKYIDQ E
Sbjct  739    EKEEKESEDKPEIEDVGSDEEEEXKKDGDKKKKKKKKKKIKEKYIDQGE

Query  290    LNKTKPIWTRNPDDITNEEYGEFYKSLTNDWEDHLAVKHFSVEGQLEFR
              LN TKPI TR PDD+TN EY EFYKSLT +WED+LAVKHFSVEGQLEFR
Sbjct  886    LN!TKPICTRYPDDFTNXEYREFYKSLTINWEDYLAVKHFSVEGQLEFR

Query  339    ALLFVPRRAPFDLFENRKKKNNIKLYVRRVFIMDNCEELIPEYLNFIRG
              A LFVPR APF+L+E RKKKN IKL  RR +IMDNCEELIPEYLNFIRG
Sbjct 1032    AFLFVPRLAPFELLETRKKKNKIKLSARRDLIMDNCEELIPEYLNFIRG

Query  388    VVDSEDLPLNISREMLQQSKILKVIRKNLVKKCLELFTELAEDKENYKK
              VVDSEDLPLNI REMLQ SKILKVIR NLVKKCLELFTEL E KE Y K
Sbjct 1179    VVDSEDLPLNIFREMLQXSKILKVIRNNLVKKCLELFTELVEGKERYXK

Query  437    FYEQFSKNIKLGIHEDSQNRKKLSELLRYYTSASGDEMVSLKDYCTRMK
              FYE FSKNIKLGIH DSQN+KKLSELLRYYT ASGDEMV L+DYCTRMK
Sbjct 1326    FYEPFSKNIKLGIHGDSQNQKKLSELLRYYTYASGDEMVYLQDYCTRMK

Query  486    ENQKHIYYITGETKDQVANSAFVERLRKHGLEVIYMIEPIDEYCVQQLK
              ENQKHIYYITGETKDQVANS +V+RL KHGLEVIY IEPIDEYCVQQLK
Sbjct 1473    ENQKHIYYITGETKDQVANSTIVQRLWKHGLEVIYTIEPIDEYCVQQLK

Query  535    EFEGKTLVSVTKEGLELPEDEEEKKKQEE-------KKTKFENLCKIMK
              EFEGKTLVSVTKE LELPEDEEEKKKQEE       KK KFENLCKI+K
Sbjct 1620    EFEGKTLVSVTKEDLELPEDEEEKKKQEEGKQKTKQKK!KFENLCKIVK
```

```
Query    577  DILEKKVEKVVVSNRLVTSPCCIVTSTYGWTANMERIMKAQALRDNSTM
              DILEK VEKVVVSN+LVTSPCCIV+STYGWTANMERIMKAQALRDNST
Sbjct   1769  DILEK!VEKVVVSNQLVTSPCCIVSSTYGWTANMERIMKAQALRDNSTT

Query    626  GYMAAKKHLEINPDHSIIETLRQKAEADKNDKSVKDLVILLYETALLSS
              GYMAAKKHLEINPDHS+I+TLRQKAE DKNDKSVKDLVILLYETALLSS
Sbjct   1914  GYMAAKKHLEINPDHSFIDTLRQKAETDKNDKSVKDLVILLYETALLSS

Query    675  GFSLEDPQTHANRIYRMIKLGLGIDEDDPTADDTSAAVTEEMPPLEGDD
               F LE+PQTHANRIYRM KLGLG DEDDPTADDTSAAVTEEMPPLEGDD
Sbjct   2061  DFGLEGPQTHANRIYRMNKLGLGTDEDDPTADDTSAAVTEEMPPLEGDD

Query    724  DTSRMEE
              DTSRME+
Sbjct   2208  DTSRMEK
```

HSP90AA5P

```
Query    192  EDQTEYLEERRIKEIVKKHSQFIGYPITLFVEKERDKEVSDDEAEEKED
              E + EYLEERRIKEIVKKHSQFIGYPITLFVEK+R+K+VSD EAE+KED
 Sbjct    22  ERRPEYLEERRIKEIVKKHSQFIGYPITLFVEKKRNKQVSDAEAEKKED

Query    241  KEEEKEKEEEKESEDKPEIEDVGSDEEEEKKDGDKKKKKKIKEKYIDQEE
              K   K++  KES DKPEIEDVGSDEEEEKKD DKKKKK  KEKYIDQE
Sbjct    169  ----KRKKKKESNDKPEIEDVGSDEEEEKKDADKKKKKKS-KEKYIDQE-

Query    290  LNKTKPIWTRNPDDITNEEYGEFYKSLTNDWEDHLAVKHFSVEGQLE
              LNKTKPIWTRNPD ITNEEYGEF++SLTN+WEDHLAVKHFSVEGQLE
Sbjct    298  LNKTKPIWTRNPDAITNEEYGEFHQSLTNNWEDHLAVKHFSVEGQLE

Query    337                            RALLFVPRRAPFDLFENRKKKNN
                                        RA LFV R APFDLFENRKKKNN
Sbjct    439   <2-----[441  :  465]-2>  RA-LFVSRCAPFDLFENRKKKNN

Query    361  IKLYVRRVFIMDNCEELIPEYLNFIRGVVDSEDLPLNISREMLQQSKIL
              I+LYV RVFIM NCEELIPEYLNFIRGV+DSEDLPLNI  EMLQQSKIL
Sbjct    533  IQLYVHRVFIMGNCEELIPEYLNFIRGVMDSEDLPLNIFSEMLQQSKIL

Query    410  KVIRKNLVKKCLELFTELAEDKENYKKFYEQFSKNIKLGIHEDSQNRKK
              KVIRKNLVK  LELF ELAEDK+NYKKF+E++S+NI L   + DSQN+KK
Sbjct    680  KVIRKNLVKTYLELFAELAEDKKNYKKFHEKLSENINL!EYIDSQNQKK

Query    459  LSELLRYYTSASGDEMVSLKDYCTRMKENQKHIYYITGETKDQVANSAF
              LSELL+   TSAS DEMV L   +   RMK NQKHIYYITGETKDQVANSAF
Sbjct    825  LSELLKXCTSASRDEMVYLIVF-SRMKANQKHIYYITGETKDQVANSAF
```

```
Query    508  VERLRKHGLEVIYMIEPIDEYCVQQLKEFEGKTLVSVTKEGLELPEDEE
              VE L+KHGLEVIYMIE ID+YCVQQLKE+E KT+VSV KEGLELPEDEE
Sbjct    969  VECLQKHGLEVIYMIELIDKYCVQQLKELESKTVVSVAKEGLELPEDEE

Query    557  EKKKQEEKKTKFENLCKIMKDILEKKVEKVVVSNRLVTSPCCIVTSTYG
              EKKKQEEKKTKFENLCKIMKD+LEKKV+KVVVSN  VTSPCCIVTSTYG
Sbjct   1116  EKKKQEEKKTKFENLCKIMKDMLEKKVKKVVVSN!-VTSPCCIVTSTYG

Query    606  WTANMERIMKAQALRDNSTMGYMAAKKHLEINPDHSIIETLRQKAEADK
              WTANMERIMKAQA  DNSTMGY+AAKKHLEINPDHS IETLRQKAEADK
Sbjct   1259  WTANMERIMKAQARXDNSTMGYVAAKKHLEINPDHS!IETLRQKAEADK

Query    655  NDKSVKDLVILLYETALLSSGFSLEDPQTHANRIYRMIKLGLGIDEDDP
              N+KSVKDLVILLY TAL S GFS+EDPQ H N+IYRMIKLGLG+DE DP
Sbjct   1405  NNKSVKDLVILLYXTALPSPGFSMEDPQRHTNKIYRMIKLGLGVDEYDP

Query    704  TADDTSAAVTEEMPPLEGDDDTSRMEEV
              TA+D +AA+T+EMPPL G DDTSRMEEV
Sbjct   1552  TANDINAAITKEMPPLRGGDDTSRMEEV
```

CSMD3

```
Query      1  MGVDIRHNKDRKVRRKEPKSQDIYLRLLV-KLYRFLARRTNSTFNQVVL
              MGVD RH KDRKVRRKEPKSQDIYLRL V     +  ++ TNSTFN+VVL
Sbjct      1  MGVDTRH-KDRKVRRKEPKSQDIYLRLSVGQAVQVSSKGTNSTFNRVVL

Query     49  KRLFMSRTNRPPLSLSRMIRKMKLPGRENKTAVVVGTITDDVRVQEVPK
              KRLFMSR NRPPL LSRMI KMKLPGRENKTAVVVG ITDDVRVQEVPK
Sbjct    145  KRLFMSRNNRPPLPLSRMIWKMKLPGRENKTAVVVGAITDDVRVQEVPK

Query     98  LKVCALRVTSRARSRILR-------------AGGKILTFDQLALDSPKG
              LKVCALR+TSRARS I               A GK LTF QLALDSPKG
Sbjct    292  LKVCALRMTSRARSHIFXGVGWGGGGAERSGARGKSLTFRQLALDSPKG

Query    134  CGTVLLSGPRKGREVYRHFGKAPGTPHSHTKPYVRSKGRKFERARGRRA
              CG V LSGP KGR VY         P +  KPY+RS GRKFERAR RRA
Sbjct    439  CGVVRLSGPPKGRGVY--------APET!CKPYIRSNGRKFERARDRRA

Query    183  SRGYKN
              SRGYKN
Sbjct    560  SRGYKN
```

WBP2NL

```
Query    1  MTDAAVSFAKDFLAGGVAAAISKTAVAPIERVKLLLQVQHASKQITADK
            +T+AAVSFAKD LAGGVAAAISK AVAPIERVKLLLQVQHASKQITADK
Sbjct    1  ITEAAVSFAKDSLAGGVAAAISKMAVAPIERVKLLLQVQHASKQITADK


Query   50  QYKGIIDCVVRIPKEQGVLSFWRGNLANVIRYFPTQALNFAFKDKYKQI
            QYKGI DC+V IPKE GVLS W GNLA+VIRYFP QALNF FKDKYKQI
Sbjct  148  QYKGIRDCMVCIPKEHGVLS-WCGNLASVIRYFP!QALNFTFKDKYKQI


Query   99  FLGGVDKRTQFWLYFAGNLASGGAAGATSLCFVYPLDFARTRLAADVGK
            FL GVDKR+QFW YFAGNLASGGA GATSLCFVYPLDFA TR+AADVGK
Sbjct  293  FLDGVDKRSQFWRYFAGNLASGGATGATSLCFVYPLDFAHTRVAADVGK


Query   48  AGAEREFRGLGDCLVKIYKSDGIKGLYQGFNVSVQGIIIYRAA---YFG
            AGAERE+RG GDCLVKIYKSDGIK LYQGFNVSVQGII YRAA   YFG
Sbjct  440  AGAERELRGFGDCLVKIYKSDGIKSLYQGFNVSVQGII-YRAACC!YFG


Query  194  IYDTAKGMLPDPKNTHIVISWMIAQTVTAVAGLTSYPFDTVRRRMMMQS
             YDTAKGMLPDPKNTHIVISWM AQTVTAVAGLTSY F+T+   MMMQS
Sbjct  582  NYDTAKGMLPDPKNTHIVISWM!AQTVTAVAGLTSYSFNTICCYMMMQS


Query  243  GRKGTDIMYTGTLDCWRKIARDEGGKAFFKGAWSNVLRGMGGAFVLVLY
            G KGTDIMYTGT DCWRKI  DEG KA FKGAWS+VLRGMGGAFVLVLY
Sbjct  728  GCKGTDIMYTGTFDCWRKITHDEGSKAVFKGAWSSVLRGMGGAFVLVLY


Query  292  DEIKKY
            DE KKY
Sbjct  875  DETKKY
```

AC019016.1

```
Query    9  AEFIVGGKYKLVRKIGSGSFGDIYLAINITNGEEVAVKLESQKARHPQL
            AEFIVG KYKL+ KI   SFGDI LAI+ITNGEEV VK ESQ ARHPQL
Sbjct  393  AEFIVGRKYKLLWKIWCSSFGDICLAISITNGEEVVVKPESQ!ARHPQL


Query   58  LYESKLYKILQGGVGIPHIRWYGQEKDYNVLVMDLLGPSLEDLFNFCSR
            LY+S+LYK+LQGGVGIPHIRW GQE+DYNVLVMD  G  L    NFCSR
Sbjct  538  LYKSRLYKMLQGGVGIPHIRWCGQEEDYNVLVMDFSG!-LASKTNFCSR


Query  107  RFTMKTVLMLADQMISRIEYVHTKNFIHRDIKPDNFLMGIGRHCNKLFL
            RFTMKTVL+LADQMISRIE+VHTKNFIHR IKP NFLMGIGRHC+KLF
Sbjct  681  RFTMKTVLILADQMISRIEHVHTKNFIHRAIKPGNFLMGIGRHCSKLFR


Query  156  IDFGLAKKYRDNRTQHIPYREDKNLTGTARYASINAHLGIEQSRRDDM
            IDFGLAK Y DNRTQHIPYREDKNLTGTA YAS  AHL IEQSRRDDM
Sbjct  828  IDFGLAKTYGDNRTQHIPYREDKNLTGTALYAS-HAHLSIEQSRRDDM
```

```
Query    205  ESLGYVLMYFNRTS---LPWQGLKAATKKQKYEKISEKKMSTPVEVLCK
              ESL YVLMYFNRTS   LPWQGLKAA +KQKYEKISEKKMSTPV   CK
Sbjct    972  ESLEYVLMYFNRTSQP!LPWQGLKAAAEKQKYEKISEKKMSTPV--FCK

Query    251  GFPAEFAMYLNYCRGLRFEEAPDYMYLRQLFRILFRTLNHQYDYTFDWT
              GF AEFA+YLN CRGL +E+APDYMYL QLF ILFRTL HQYDYTFDWT
Sbjct   1112  GFAAEFAIYLNPCRGLHLEKAPDYMYLTQLFCILFRTLIHQYDYTFDWT

Query    300  MLKQKAAQQAASSSGQGQQAQTPTGKQTDKTKSNMKGF
               LKQKAAQQAASSSG GQQAQTPTGKQ DKTK NMKGF
Sbjct   1259  KLKQKAAQQAASSSG!GQQAQTPTGKQPDKTKGNMKGF
```
