# Supplementary material for: Newly evolved introns in human retrogenes provide novel insights into their evolutionary roles
Source: BMC Evol Biol. 2012 Jul 28;12:128. doi: 10.1186/1471-2148-12-128 (PMC3565874; doi:10.1186/1471-2148-12-128)
Supplement: Additional file 11 — Nucleotide-level alignments of retrogene introns (‘Sbjct’, blue and red, splice sites) and parental genes (‘Query’, program NCBI-BLAST). This file contains alignments of intron-gain retrogens and their parents in DNA level [file 1471-2148-12-128-S11.pdf]

Additional file 11

Nucleotide-level alignments of retrogene introns (‘Sbjct’, blue and red, splice sites) and parental genes (‘Query’, program NCBI-BLAST [S1]).

Red downward arrows indicate the exon-exon borders of parental genes. In XXyac-R12DG2.2, the retrogene is also annotated to be embedded near the 3’ end of an ncRNA gene candidate (LOC100190939, Additional file 12). In CSMD3, green mark the intragenic region of the retroposed sequnece. The retrogene is an alternative translation start point (Additional file 12). In WBP2NL, the retrogene is located in its first intron, but could be transcribed at sometimes (Additional file 1 and 2). Besides, the intronic region at XXyac-R12DG2.2 and AC019016.1 could be spliced in two patterns.

References

S1. Johnson M, Zaretskaya I, Raytselis Y, Merezhuk Y, McGinnis S, Madden TL: **NCBI BLAST: a better web interface**. *Nucleic Acids Res* 2008, **36 (Web Server issue)**:W5-9.

TMEM14D

```
Query     ACAGGCAGCGTGCCGTCCCTGGCTGCAGGGCTGCTCTTCGGCAGTCTAGCCGGCCTGGGT
          |||||||| | ||||||||||||||||||||||||||| ||||||||||||||| |||||
Sbjct     ACAGGCAGGGCGCCGTCCCTGGCTGCAGGGCTGCTCTTTGGCAGTCTAGCCGGCGTGGGT

Query     GCTTACCAGCTGTATCAGGATCCAAGGAACGTTTGGGGTTTCCTAGCCGCTACATCTG
          ||||||||||||||||||||||||||||| ||||||| ||||||||||||||||||||
Sbjct     GCTTACCAGCTGTATCAGGATCCAAGGAATGTTTGGGATTTCCTAGCCGCTACATCTG
```

RPS3AP5

```
Query     TGGTTTTACTAAAAAACGCAACAATCAGATACGGAAGACCTCTTATGCTCAGCACCAACA
          ||||||||||||||||||||||||||||||||||||||||||||||||||||||||| ||
Sbjct     TGGTTTTACTAAAAAACGCAACAATCAGATACGGAAGACCTCTTATGCTCAGCACCAGCA

Query     GGTCC-----------------------------------GCCAAATCCGGAAGAAGATG
          |||||                                   ||||||||||||||||||||
Sbjct     GGTCCTGAGAAATCCGGAAGACCTCTTATGCTCAGTACCAGCCAAATCCGGAAGAAGATG

Query     ATGGAAATCATGACCCGAGAGGTGCAGACAAATGACTTGAAAGAAGTGGTCAATAAATTG
          ||||||||||||||||||||||||||||||||||||||||||||||||||||||||||||
Sbjct     ATGGAAATCATGACCCGAGAGGTGCAGACAAATGACTTGAAAGAAGTGGTCAATAAATTG
```

XXyac-R12DG2.2

```
Query     GACAATGATGGGGATGGCTTTGTCACTACTGAGGAGCTGAAAACCTGGATCAAACGGGTG
          |||||||||||| |||||||||||||||||||||||||||||||||||||||||||||||
Sbjct     GACAATGATGGGTATGGCTTTGTCACTACTGAGGAGCTGAAAACCTGGATCAAACGGGTG

Query     CAGAAAAGATACATCTTTGATAATGTCGCCAAAGTCTGGAAGGATTATGATAGGGACAAG
          ||||||||||||||||||||||||||||||||||||||||||||||||||||||||||||
Sbjct     CAGAAAAGATACATCTTTGATAATGTCGCCAAAGTCTGGAAGGATTATGATAGGGACAAG

Query     GATGATAAAATTTCCTGGGAAGAATACAAACAAGCCACCTATGGTTAC--TACCTAGGAA
          |||||||| |||||||||||||||||||||||||||||||||||||||  ||||||||||
Sbjct     GATGATAACATTTCCTGGGAAGAATACAAACAAGCCACCTATGGTTACTATACCTAGGAA

Query     ACCCCGCAGAGTTTCATGATTCTTCAGATCATCACACCTTTAAAAAGATGCTGCCACGTG
          ||||||||||||||||||||||||||||||||||||||||||||||||||||||||||||
Sbjct     ACCCCGCAGAGTTTCATGATTCTTCAGATCATCACACCTTTAAAAAGATGCTGCCACGTG
```

HSP90B2P

```
Query     TCTTGCTAAACTTCTTAGGTTCCAGTCTTCTCATCATCCAACTGACATTACTAGCCTAGA
          |||||||||||||||||||||||||||||||||||||||| ||||||||||||||||| |
Sbjct     TCTTGCTAAACTTCTTAGGTTCCAGTCTTCTCATCATCCAGCTGACATTACTAGCCTACA

Query     CCAGTATGTGGAAAGAATGAAGGAAAAACAAGACAAAATCTACTTCATGGCTGGGTCCAG
          |||| |||| ||||||||||||||||||||||||||||||| | ||||||||||||||||
Sbjct     CCAGGATGTTGAAAGAATGAAGGAAAAACAAGACAAAATCTGCCTCATGGCTGGGTCCAG

Query     CAGAAAAGAGGCTGAATCTTCTCCATTTGTTGAGCGACTTCTGAAAAAGGGCTATGAAGT
          ||||||||||||||||||||||||||||||| ||||||||||||||| ||||||||||||
Sbjct     CAGAAAAGAGGCTGAATCTTCTCCATTTGTTTAGCGACTTCTGAAAAGGGGCTATGAAGT
```

```
Query      TATTTACCTCACAGAACCTGTGGATGAATACTGTATTCAGGCCCTTCCCGAATTTGATGG
           ||||||||||||||||||||||| ||||||||| ||||||||||||||||||||||||||
Sbjct      TATTTACCTCACAGAACCTGTGGTTGAATACTGCATTCAGGCCCTTCCCGAATTTGATGG

Query      GAAGAGGTTCCAGAATGTTGCCAAGGAAGGAGTGAAGTTCGATGAAAGTGAGAAAACTAA
           ||||||||||||||||||||||||||||||||||||||| ||||| ||||||||||||||
Sbjct      GAAGAGGTTCCAGAATGTTGCCAAGGAAGGAGTGAAGTTTGATGACAGTGAGAAAACTAA

Query      GGAGAGTCGTGAAGCAGTTGAGAAAGAATTTGAGCCTCTGCTGAATTGGATGAAAGATAA
           |||||||| ||||||||||||||||||||||||||||||||  |||||| ||||||||||
Sbjct      GGAGAGTCATGAAGCAGTTGAGAAAGAATTTGAGCCTCTGCCCAATTGGGTGAAAGATAA

Query      AGCCCTTAAGGACAAGATTGAAAAGGCTGTGGTGTCTCAGCGCCTGACAGAATCTCCGTG
           |||| ||||||||||||||||||||||| |||| |||||| ||||||||||||||| |||
Sbjct      AGCCATTAAGGACAAGATTGAAAAGGCTATGGTATCTCAGTGCCTGACAGAATCTCTGTG

Query      TGCTTTGGTGGCCAGCCAGTACGGATGGTCTGGCAACATGGAGAGAATCATGAAAGCACA
           |||||||||||||||||||||||| |||||||||||||||||||||||||||||||||||
Sbjct      TGCTTTGGTGGCCAGCCAGTACGGGTGGTCTGGCAACATGGAGAGAATCATGAAAGCACA

Query      AGCGTACCAAACGGGCAAGGACATCTCTACAAATTACTATGCGAGTCAGAAGAAAACATT
           |||||||||||||||||||| |||||||||||||||| ||||||||| ||||||||||||
Sbjct      AGCGTACCAAACGGGCAAGGGCATCTCTACAAATTACCATGCGAGTCGGAAGAAAACATT

Query      TGAAATTAATCCCAGACACCCGCTGATCAGAGACATGCTTCGACGAATTAAGGAAGATGA
           |||||||||||||||||| |||||||||||||||||||||||||||||||||||||||||
Sbjct      TGAAATTAATCCCAGACATCCGCTGATCAGAGACATGCTTCGACGAATTAAGGAAGATGA

Query      AGATGATAAAACAGTTTTGGATCTTGCTGTGGTTTTGTTTGAAACAGCAACGC-------
           |||||||||||||||||||||||||||||||||||||||||||||||||| ||
Sbjct      AGATGATAAAACAGTTTTGGATCTTGCTGTGGTTTTGTTTGAAACAGCAATGCAGCAGTG

Query      -TTCGGTCAGGGTATCTTTTACCAGACACTAAAGCATATGGAGATAGAATAGAAAGAATG
            |||||||||| | ||||||||||||||||||||||||||||||||||||||||||||||
Sbjct      ATTCGGTCAGGATGTCTTTTACCAGACACTAAAGCATATGGAGATAGAATAGAAAGAATG

Query      CTTCGCCTCAGTTTGAACATTGACCCTGATGCAAAGGTGGAAGAAGAGCCCGAAGAAGAA
           | | |||||||||||||||   | |||||||||||||||||||||   ||||| ||||||
Sbjct      CCTTGCCTCAGTTTGAACA--CATCCTGATGCAAAGGTGGAAGAA---CCCGACGAAGAA

Query      CCTGAAGAGACAGCAG
           ||||| ||||||||||
Sbjct      CCTGAGGAGACAGCAG
```

HSP90AA4P

```
Query      TGGGAGTCCTCAGCAGGGGGATCATTCACAGTGAGGACAGACACAGGTGAACCTATGGGT
           ||||||||||||  | |||||||||||||||||||||||||||||||||||||||| |||
Sbjct      TGGGAGTCCTCATTAAGGGGATCATTCACAGTGAGGACAGACACAGGTGAACCTATAGGT

Query      CGTGGAACAAAAGTTATCCTACACCTGAAAGAAGACCAAACTGAGTACTTGGAGGAACGA
           | ||||||||| ||||||||||  || |||||||||||||||||||||||||||||||||
Sbjct      CATGGAACAAAGGTTATCCTACCGCTAAAAGAAGACCAAACTGAGTACTTGGAGGAACGA

Query      AGAATAAAGGAGATTGTGAAGAAACATTCTCAGTTTATTGGATATCCCATTACTCTTTTT
           | |||||| |||||||||||||||||||||  |||||||||||||||| | |||||||||
Sbjct      AAAATAAATGAGATTGTGAAGAAACATTCT--GTTTATTGGATATCCCGTCACTCTTTTT
```

```
Query     GTGGAGAAGGAACGTGATAAAGAAGTAAGCGATGATGAGGCTGAAGAAAAGGAAGACAAA
          ||||||||| ||| |||||||||||| ||| |||||||||| |||||||||||||| |||
Sbjct     GTGGAGAAGAAACATGATAAAGAAGTCAGCAATGATGAGGCCGAAGAAAAGGAAGATAAA

Query     GAAGAAGAAAAAGAAAAAGAAGAGAAAGAGTCGGAAGACAAACCTGAAATTGAAGATGTT
          ||||||||||| |||||||||||||||||||| |||||||||||||||||||||||||||
Sbjct     GAAGAAGAAAAGGAAAAAGAAGAGAAAGAGTCCGAAGACAAACCTGAAATTGAAGATGTT

Query     GGTTCTGATGAGGAAGAAGAA---AAGAAGGATGGTGACAAGAAGAAGAAGAAGAAGA--
          |||||||| || |||||||||   ||||||||||||||||||||||||||||||||||  
Sbjct     GGTTCTGACGAAGAAGAAGAATAAAAGAAGGATGGTGACAAGAAGAAGAAGAAGAAGAAG

Query     ----------TTAAGGAAAAGTACATCGATCAAGAAGAGCTCAACAAAACAAAGCCCATC
                    |||||||||||||||||||||||| ||| ||||||||| ||||||| |||
Sbjct     AAGAAGAAGATTAAGGAAAAGTACATCGATCAAGGAGAACTCAACAAA-CAAAGCCTATC

Query     TGGACCAGAAATCCCGACGATATTACTAATGAGGAGTACGGAGAATTCTATAAGAGCTTG
          || |||||| | || || ||  |||||||| |||| ||| |||||||||| || ||||||
Sbjct     TGTACCAGATACCCTGATGACTTTACTAATTAGGAATACAGAGAATTCTACAAAAGCTTG

Query     ACCAATGACTGGGAAGATCACTTGGCAGTGAAGCATTTTTCAGTTGAAGGACAGTTGGAA
          |||| | ||||||||||| |||||||||||||||||||||||||||||||||||||||||
Sbjct     ACCATTAACTGGGAAGATTACTTGGCAGTGAAGCATTTTTCAGTTGAAGGACAGTTGGAA

Query     TTCAGAGCCCTTCTATTTGTCCCACGACGTGCTCCTTTTGATCTGTTTGAAAACAGAAAG
          ||||||||| |||||||||||||||||| |||||||||||| ||||| |||| |||||||
Sbjct     TTCAGAGCCTTTCTATTTGTCCCACGACTTGCTCCTTTTGAGCTGTTGGAAACCAGAAAG

Query     AAAAAGAACAACATCAAATTGTATGTACGCAGAGTTTTCATCATGGATAACTGTGAGGAG
          ||||||||||| |||||||||| || |||||||| | |||||||||||||||||||||||
Sbjct     AAAAAGAACAAAATCAAATTGTCTGCACGCAGAGATCTCATCATGGATAACTGTGAGGAG

Query     CTAATCCCTGAATATCTGAACTTCATTAGAGGGGTGGTAGACTCGGAGGATCTCCCTCTA
          |||||||||||||||||||||||||| |||||||||||||||||||||||||||||||||
Sbjct     CTAATCCCTGAATATCTGAACTTCATCAGAGGGGTGGTAGACTCGGAGGATCTCCCTCTA

Query     AACATATCCCGTGAGATGTTGCAACAAAGCAAAATTTTGAAAGTTATCAGGAAGAATTTG
          || || | ||||||||||||||||  ||||||||||||||||||||||||||| ||||||
Sbjct     AATATTTTCCGTGAGATGTTGCAATGAAGCAAAATTTTGAAAGTTATCAGGAATAATTTG

Query     GTCAAAAAATGCTTAGAACTCTTTACTGAACTGGCGGAAGATAAAGAGAACTACAAGAAA
          ||||||||||||||||||||||||||||||||||  |||| ||||||||  ||| |||| 
Sbjct     GTCAAAAAATGCTTAGAACTCTTTACTGAACTGGTAGAAGGTAAAGAGAGGTACTAGAAG

Query     TTCTATGAGCAGTTCTCTAAAAACATAAAGCTTGGAATACACGAAGACTCTCAAAATCGG
          |||||||||| |||||||||||||||||||||||||||||||| |||||||||||||| |
Sbjct     TTCTATGAGCCGTTCTCTAAAAACATAAAGCTTGGAATACACGGAGACTCTCAAAATCAG

Query     AAGAAGCTTTCAGAGCTGTTAAGGTACTACACATCTGCCTCTGGTGATGAGATGGTTTCT
          ||||| |||||||||||| |||| |||||||||| ||||||||||||||||||||||| |
Sbjct     AAGAAACTTTCAGAGCTGCTAAGATACTACACATATGCCTCTGGTGATGAGATGGTTTAT

Query     CTCAAGGACTACTGCACCAGAATGAAGGAGAACCAGAAACATATCTATTATATCACAGGT
          ||| ||||||||||||||||||||||||| ||||||||||||||||||||||||||||||
Sbjct     CTCCAGGACTACTGCACCAGAATGAAGGAAAACCAGAAACATATCTATTATATCACAGGT
```

```
Query  GAGACCAAGGACCAGGTAGCTAACTCAGCCTTTGTGGAACGTCTTCGGAAACATGGCTTA
       ||||||||||||||||||||||||||| || ||||| |||||||| ||||||||||||| 
Sbjct  GAGACCAAGGACCAGGTAGCTAACTCAACCATTGTGCAACGTCTTTGGAAACATGGCTTG

Query  GAAGTGATCTATATGATTGAGCCCATTGATGAGTACTGTGTCCAACAGCTGAAGGAATTT
       ||||||||||||| ||| |||||||||||||||||||||||||| |||||||||||||||
Sbjct  GAAGTGATCTATACGATCGAGCCCATTGATGAGTACTGTGTCCAGCAGCTGAAGGAATTT

Query  GAGGGGAAGACTTTAGTGTCAGTCACCAAAGAAGGCCTGGAACTTCCAGAGGATgaagaa
       |||||||||||||||||||||||||||||||| | | |||||||||||||||||||||||
Sbjct  GAGGGGAAGACTTTAGTGTCAGTCACCAAAGAGGACTTGGAACTTCCAGAGGATGAAGAA

Query  GAGAAAAAGAAGCAGGAAGAG-----------------------AAAAAAACAAAGTTTG
       ||||||||||| |||||||||                       |||||| |||||||||
Sbjct  GAGAAAAAGAAACAGGAAGAGGGAAAACAAAAAACGAAACAAAAAAAAAACCAAAGTTTG

Query  AGAACCTCTGCAAAATCATGAAAGACATATTGGAGAAAAAAGTTGAAAAGGTGGTTGTGT
       ||||||||||||||||| |||||||||| ||||||  || ||||||||||||||||||||
Sbjct  AGAACCTCTGCAAAATCGTGAAAGACATTTTGGAG--AAGAGTTGAAAAGGTGGTTGTGT

Query  CAAACCGATTGGTGACATCTCCATGCTGTATTGTCACAAGCACATATGGCTGGACAGCAA
       |||||| ||||||||| |||||||| ||||||||| ||||||||||||||||||||||||
Sbjct  CAAACCAATTGGTGACGTCTCCATGTTGTATTGTCTCAAGCACATATGGCTGGACAGCAA
```

HSP90AA5P

```
Query  GGAGTACGGAGAATTCTATAAGAGCTTGACCAATGACTGGGAAGATCACTTGGCAGTGAA
       |||||| ||||| ||| || |||||||||||||| |||||||||||||||||||||||||
Sbjct  GGAGTATGGAGAGTTCCATCAGAGCTTGACCAATAACTGGGAAGATCACTTGGCAGTGAA

Query  GCATTTTTCAGTTGAAGGACAGTTGGAA----------------------TTCAGAGCCC
       ||| |||||||||||||| |||||||||                      || |||    
Sbjct  GCACTTTTCAGTTGAAGGGCAGTTGGAAGAGTTGAAGGACAGTCGAAGAGTTTAGAAGAG

Query  TTCTATTTGTCCCACGACGTGCTCCTTTTGATCTGTTTGAAAACAGAAAGAAAAAGAACA
       ||| |||||  || || ||||||||||||| || ||||||||||||||||||||||||| 
Sbjct  CTCTTTTTGTTTCAAGATGTGCTCCTTTTGACCTATTTGAAAACAGAAAGAAAAAGAACA

Query  ACATCAAATTGTATGTACGCAGAGTTTTCATCATGGATAACTGTGAGGAGCTAATCCCTG
       ||||  | |||||||||| ||||||||||||||||| |||||||||||||||||||||||
Sbjct  ACATTCAGTTGTATGTACACAGAGTTTTCATCATGGGTAACTGTGAGGAGCTAATCCCTG

Query  AATATCTGAACTTCATTAGAGGGGTGGTAGACTCGGAGGATCTCCCTCTAAACATATCCC
       |||| ||||||||||||||||||||| | ||||| ||||||||||||||||| || |   
Sbjct  AATACCTGAACTTCATTAGAGGGGTGATGGACTCAGAGGATCTCCCTCTAAATATTTTTT

Query  GTGAGATGTTGCAACAAAGCAAAATTTTGAAAGTTATCAGGAAGAATTTGGTCAAAAAAT
       |||||||||||||||||||||||||||||||||||| ||||||||||||||||||| || 
Sbjct  CTGAGATGTTGCAACAAAGCAAAATTTTGAAAGTTATTAGGAAGAATTTGGTCAAAACAT

Query  GCTTAGAACTCTTTACTGAACTGGCGGAAGATAAAGAGAACTACAAGAAATTCTATGAGC
       ||||||||||||| |||||||||| || |||||| |||||||||| || ||| | |||  
Sbjct  ACTTAGAACTCTTTGCTGAACTGGCAGAGGATAAAAAGAACTACAAAAAGTTCCACGAGA

Query  AGTTCTCTAAAAACATAAAGCTTGGAATACACGAAGACTCTCAAAATCGGAAGAAGCTTT
       |||| ||| |||||||||| ||| ||||| |   |||||||||||||| |||||||||||
Sbjct  AGTTATCTGAAAACATAAATCTTTGAATATAT--AGACTCTCAAAATCAGAAGAAGCTTT
```

```
Query    CAGAGCTGTTAAGGTACTACACATCTGCCTCTGGTGATGAGATGGTTTCTCTCAAGGACT
         |||||||||| |  || | ||||||||||||| ||||||||||||||| |||||  |  |
Sbjct    CAGAGCTGTTGAAATAGTGCACATCTGCCTCTCGTGATGAGATGGTTTATCTCATTGTGT

Query    ACTGCACCAGAATGAAGGAGAACCAGAAACATATCTATTATATCACAGGTGAGACCAAGG
           |   ||||||||||||  |||||||||||||||||||| || ||||||||||||||||
Sbjct    TTT---CCAGAATGAAGGCAAACCAGAAACATATCTATTACATTACAGGTGAGACCAAGG

Query    ACCAGGTAGCTAACTCAGCCTTTGTGGAACGTCTTCGGAAACATGGCTTAGAAGTGATCT
         |||| |||||||||||||||||||||||| |||||| ||| |||||||||||||||||||
Sbjct    ACCAAGTAGCTAACTCAGCCTTTGTGGAATGTCTTCAGAAGCATGGCTTAGAAGTGATCT

Query    ATATGATTGAGCCCATTGATGAGTACTGTGTCCAACAGCTGAAGGAATTTGAGGGGAAGA
         ||||||| |||   |||||| ||||||||||||| |||||||||||| ||||| | ||||
Sbjct    ATATGATCGAGTTGATTGATAAGTACTGTGTCCAGCAGCTGAAGGAACTTGAGAGTAAGA

Query    CTTTAGTGTCAGTCACCAAAGAAGGCCTGGAACTTCCAGAGGATGAAGAAGAGAAAAAGA
         || ||||||||||  ||||||| ||  ||||||||||||| ||||||||||| |||||||
Sbjct    CTGTAGTGTCAGTTGCCAAAGAGGGTTTGGAACTTCCAGAAGATGAAGAAGAAAAAAAGA

Query    AGCAGGAAGAGAAAAAAACAAAGTTTGAGAACCTCTGCAAAATCATGAAAGACATATTGG
         | ||||||||||||||||||||||||||||||||||||||||||||||||||||| ||||
Sbjct    AACAGGAAGAGAAAAAAACAAAGTTTGAGAACCTCTGCAAAATCATGAAAGACATGTTGG

Query    AGAAAAAAGTTGAAAAGGTGGTTGTGTCAAACCGATTGGTGACATCTCCATGCTGTATTG
         ||||||||||  ||||||||||||||||||||    |||||||||| |||||||||||||
Sbjct    AGAAAAAAGTCAAAAAGGTGGTTGTGTCAAAC----TGGTGACATCCCCATGCTGTATTG

Query    TCACAAGCACATATGGCTGGACAGCAAACATGGAGAGAATCATGAAAGCTCAAGCCCTAA
         |||||||||||||||||||||||||||||||||| ||||||||||||||||||||||
Sbjct    TCACAAGCACATATGGCTGGACAGCAAACATGGAAAGAATCATGAAAGCTCAAGCCCGGT

Query    GAGACAACTCAACAATGGGTTACATGGCAGCAAAGAAACACCTGGAGATAAACCCTGACC
         |||||||||||||||||| ||| ||||||||||||||||||||||||||||||||||||
Sbjct    AAGACAACTCAACAATGGGCTACGTGGCAGCAAAGAAACACCTGGAGATAAACCCTGACC

Query    ATTCCATTATTGAGACCTTAAGGCAAAAGGCAGAGGCTGATAAGAACGACAAGTCTGTGA
         ||| ||| ||||| ||||||||||||||||||||||||||||||||| ||||||| ||||
Sbjct    ATT-CATGATTGAAACCTTAAGGCAAAAGGCAGAGGCTGATAAGAACAACAAGTCAGTGA

Query    AGGATCTGGTCATCTTGCTTTATGAAACTGCGCTCCTGTCTTCTGGCTTCAGTCTGGAAG
         ||||||||||||||||||||||  ||||||| ||||  ||| ||||||||||| ||||||
Sbjct    AGGATCTGGTCATCTTGCTTTACTAAACTGCACTCCCATCTCCTGGCTTCAGTATGGAAG

Query    ATCCCCAGACACATGCTAACAGGATCTACAGGATGATCAAACTTGGTCTGGGTATTGATG
         | || |||| |||| |||||| |||||| |||||||||||||||||||||||| ||||||
Sbjct    ACCCTCAGAGACATACTAACAAGATCTATAGGATGATCAAACTTGGTCTGGGTGTTGATG
```

CSMD3

```
Query    ATGGGAGTGGACATCCGCCATAACAAGGACCGAAAGGTTCGGCGCAAGGAGCCCAAGAGC
         |||||||| |||| ||||||   |||||||||||||||||||||||||||||| ||||||
Sbjct    ATGGGAGTTGACACCCGCCA---CAAGGACCGAAAGGTTCGGCGCAAGGAGCCTAAGAGC

Query    CAGGATATCTACCTGAGGCTGTT----GGTCAAGTTATACAGGTTTCTGGCCAGAAGAAC
         ||||||||||||||||||||||     ||||||| | ||||||||||  || | | ||||
Sbjct    CAGGATATCTACCTGAGGCTGTCTGTCGGTCAAGCTGTACAGGTTTCCAGCAA-AGGAAC
```

```
Query    CAACTCCACATTCAACCAGGTTGTGTTGAAGAGGTTGTTTATGAGTCGCACCAACCGGCC
         ||||||||||||||||| ||||||| |||||||||||||||||||||||| |||||||||
Sbjct    CAACTCCACATTCAACCGGGTTGTGCTGAAGAGGTTGTTTATGAGTCGCAACAACCGGCC

Query    GCCTCTGTCCCTTTCCCGGATGATCCGGAAGATGAAGCTTCCTGGCCGGGAAAACAAGAC
          |||||| ||||||||||||||||| ||||||||||||||||||||||||||||||| ||
Sbjct    ACCTCTGCCCCTTTCCCGGATGATCTGGAAGATGAAGCTTCCTGGCCGGGAAAACAAAAC

Query    GGCCGTGGTTGTGGGGACCATAACTGATGATGTGCGGGTTCAGGAGGTACCCAAACTGAA
          ||||||||||||||| ||||||| ||||| ||||||||||||||||| |||||||| ||
Sbjct    AGCCGTGGTTGTGGGGGCCATAACGGATGACGTGCGGGTTCAGGAGGTGCCCAAACTAAA

Query    GGTATGTGCACTGCGCGTGACCAGCCGGGCCCGCAGCCGCATCCT---------------
         ||| ||||| |||||| ||||||||||||||||||||| |||| |
Sbjct    GGTGTGTGCGCTGCGCATGACCAGCCGGGCCCGCAGCCACATCTTCTAGGGAGTGGGGTG

Query    ------------------------CAGGGCAGGGGGCAAGATCCTCACTTTCGACCAGCT
                                 | ||||  ||||||||| |||||||||   ||||||
Sbjct    GGGTGGGGGTGGGGCGGAGCGGAGCGGGGCGCGGGGCAAGAGCCTCACTTTTCGCCAGCT

Query    GGCCCTGGACTCCCCTAAGGGCTGTGGCACTGTCCTGCTCTCCGGTCCTCGCAAGGGCCG
         ||| ||||||||||| |||||||| |||   |||| ||||||||||||||  ||||||||
Sbjct    GGCGCTGGACTCCCCCAAGGGCTGCGGCGTCGTCCGGCTCTCCGGTCCTCCAAAGGGCCG

Query    AGAGGTGTACCGGCATTTCGGCAAGGCCCCAGGAACCCCGCACAGCCACACCAAACCCTA
         || |||||||               ||||| | ||||| |           |||||||||
Sbjct    AGGGGTGTAC---------------GCCCCGGAAACCCTG-----------CAAACCCTA

Query    CGTCCGCTCCAAGGGCCGGAAGTTCGAGCGTGCCAGAGGCCGACGGGCCAGCCGAGGCTA
         | |||||||||| ||||||||||||||||| || |||| |||| ||||||||||||| ||
Sbjct    CATCCGCTCCAATGGCCGGAAGTTCGAGCGCGCGAGAGACCGAAGGGCCAGCCGAGGATA

Query    CAAAAAC
         |||||||
Sbjct    CAAAAAC
```

WBP2NL

```
Query    CATCGGGTGGTGCCGCAGGGGCCACATCCCTGTGTTTTGTGTACCCTCTTGATTTTGCCC
         |||| || |||||| | |||||||||||| ||||||||||||||||||||||||||||||
Sbjct    CATCAGGCGGTGCCACTGGGGCCACATCCTTGTGTTTTGTGTACCCTCTTGATTTTGCCC

Query    GTACCCGTCTAGCAGCTGATGTGGGTAAAGCTGGAGCTGAAAGGGAATTCCGAGGCCTCG
          ||| ||| |||||||||||||||| ||||||||||||||||||||||| |||||  |||
Sbjct    ATACTCGTGTAGCAGCTGATGTGGGCAAAGCTGGAGCTGAAAGGGAATTGCGAGGTTTCG

Query    GTGACTGCCTGGTTAAGATCTACAAATCTGATGGGATTAAGGGCCTGTACCAAGGCTTTA
         |||||||||| |||||||||||||||||||| ||||||||| ||||||||||||||||||
Sbjct    GTGACTGCCTAGTTAAGATCTACAAATCTGACGGGATTAAGAGCCTGTACCAAGGCTTTA

Query    ACGTGTCTGTGCAGGGTATTATCATCTACCGA-------GCCGCCTACTTCGGTATCTAT
         ||||||||||||||||||||   |||||||||       || ||||||||||||| ||||
Sbjct    ACGTGTCTGTGCAGGGTATT---ATCTACCGAGCTGCCTGCTGCCTACTTCGGTAACTAT

Query    GACACTGCAAAGGGAATGCTTCCGGATCCCAAGAACACTCACATCGTCATCAGCTGGATG
         |||||||||||||||||||||||||||||||||||||||||||| |||||||| ||||||
Sbjct    GACACTGCAAAGGGAATGCTTCCGGATCCCAAGAACACTCACATTGTCATCAGTTGGATG
```

```
Query      ATCGCACAGACTGTCACTGCTGTTGCCGGGTTGACTTCCTATCCATTTGACACTGTTCGC
           || ||||||||||||||||| ||||| ||||||||||||||| ||||| ||||  || ||
Sbjct      AT-GCACAGACTGTCACTGCCGTTGCTGGGTTGACTTCCTATTCATTTAACACCATTTGC
```

AC019016.1

```
Query      GCGATCAACATCACCAACGGCGAGGAAGTGGCAGTGAAGCTAGAATCTCAGAAGGCCAGG
           ||||||| |||||||||||| || ||||||| |||||||| ||||||||||  |||||||
Sbjct      GCGATCAGCATCACCAACGGTGAAGAAGTGGTAGTGAAGCCAGAATCTCAG--GGCCAGG

Query      CATCCCCAGTTGCTGTACGAGAGCAAGCTCTATAAGATTCTTCAAGGTGGGGTTGGCATC
           |||||||||||||||||| |||||| ||| || ||||| |||||||||||||||||||||
Sbjct      CATCCCCAGTTGCTGTACAAGAGCAGGCTTTACAAGATGCTTCAAGGTGGGGTTGGCATC

Query      CCCCACATACGGTGGTATGGTCAGGAAAAAGACTACAATGTACTAGTCATGGATCTT-CT
           |||||||||||||||| |||||||||| ||||||| |||||||||||||||||| || ||
Sbjct      CCCCACATACGGTGGTGTGGTCAGGAAGAAGACTATAATGTACTAGTCATGGATTTTTCT

Query      GGGACCTAGCCTCGAAGACCTCTTCAATTTCTGTTCAAGAAGGTTCACAATGAAAACTGT
           ||||||||||||||||||||     |||||||||||||||||||||||||||||||||||
Sbjct      GGGACCTAGCCTCGAAGACC-----AATTTCTGTTCAAGAAGGTTCACAATGAAAACTGT

Query      ACTTATGTTAGCTGACCAGATGATCAGTAGAATTGAATATGTGCATACAAAGAATTTTAT
           |||||| ||||||||||||||||||||||||||||||| |||||||||||||||||||||
Sbjct      ACTTATATTAGCTGACCAGATGATCAGTAGAATTGAACATGTGCATACAAAGAATTTTAT

Query      ACACAGAGACATTAAACCAGATAACTTCCTAATGGGTATTGGGCGTCACTGTAATAAGTT
           |||||||| ||||||||||| |||||||||||||||||||||||||||||||| ||||||
Sbjct      ACACAGAGCCATTAAACCAGGTAACTTCCTAATGGGTATTGGGCGTCACTGTAGTAAGTT

Query      ATTCCTTATTGATTTTGGTTTGGCCAAAAAGTACAGAGACAACAGGACAAGGCAACACAT
           ||||| ||||||||||||||||||||||| |||| |||||||||||||||||||||||||
Sbjct      ATTCCGTATTGATTTTGGTTTGGCCAAAACGTACGGAGACAACAGGACAAGGCAACACAT

Query      ACCATACAGAGAAGATAAAAACCTCACTGGCACTGCCCGATATGCTAGCATCAATGCACA
           ||||||||||||||||||||||||||||||||||||||  |||||||||    |||||||
Sbjct      ACCATACAGAGAAGATAAAAACCTCACTGGCACTGCCCTGTATGCTAGCC---ATGCACA

Query      TCTTGGTATTGAGCAGAGTCGCCGAGATGACATGGAATCATTAGGATATGTTTTGATGTA
           |||| ||||||||||||||||||| |||||||||||||| |||| |||||||||||||||
Sbjct      TCTTAGTATTGAGCAGAGTCGCCGGGATGACATGGAATCGTTAGAATATGTTTTGATGTA

Query      TTTTAATAGAACCAGCC--------TGCCATGGCAAGGGCTAAAGGCTGCAACAAAGAAA
           |||||||||||||||||        |||||||||||||||||||||||||| || |||||
Sbjct      TTTTAATAGAACCAGCCAACCAGCCTGCCATGGCAAGGGCTAAAGGCTGCAGCAGAGAAA

Query      CAAAAATATGAAAAGATTAGTGAAAAGAAGATGTCCACGCCTGTTGAAGTTTTATGTAAG
           ||||||||||||||||||||||||||||||||||| || ||||||      || ||||||
Sbjct      CAAAAATATGAAAAGATTAGTGAAAAGAAGATGTCTACTCCTGTT------TTTTGTAAG

Query      GGGTTTCCTGCAGAATTTGCGATGTACTTAAACTATTGTCGTGGGCTACGCTTTGAGGAA
           |||||| ||||||||||||| || |||||||||  |||||||||||||| ||| ||| ||
Sbjct      GGGTTTGCTGCAGAATTTGCCATATACTTAAACCCTTGTCGTGGGCTACACTTAGAGAAA

Query      GCCCCAGATTACATGTATCTGAGGCAGCTATTCCGCATTCTTTTCAGGACCCTGAACCAT
           |||||||||||||||||||||| |||||||||| ||||||||||||||||||||| ||||
Sbjct      GCCCCAGATTACATGTATCTGACGCAGCTATTCTGCATTCTTTTCAGGACCCTGATCCAT
```

```
Query   CAATATGACTACACATTTGATTGGACAATGTTAAAGCAGAAAGCAGCACAGCAGGCAGCC
        |||||||||||||||||||||||||||| |||||||||||||||||||||||||||||||
Sbjct   CAATATGACTACACATTTGATTGGACAAAGTTAAAGCAGAAAGCAGCACAGCAGGCAGCC
```
