# Supplementary material for: Newly evolved introns in human retrogenes provide novel insights into their evolutionary roles
Source: BMC Evol Biol. 2012 Jul 28;12:128. doi: 10.1186/1471-2148-12-128 (PMC3565874; doi:10.1186/1471-2148-12-128)

A**dditional file 12**

**Positions of three retrogenes (XXyac-R12DG2.2, CSMD3 and WBP2NL) in the human genome (from the UCSC Genome Browser database [S1,S2]).**

The red band below the text “Your Sequence from Blat Search” is the corresponding region of the retrogene. From these snapshots, we could see that the retrogene of XXyac-R12DG2.2 is also embedded near the 3’ end of an ncRNA gene candidate, and the retrogene of CSMD3 is an alternative translation start point. In WBP2NL, the retrogene is located at the first intron, but it could be transcribed in some circumstances (Additional file 1 and 2).

**References**

S1. Karolchik D, Hinrichs AS, Furey TS, Roskin KM, Sugnet CW, Haussler D, Kent WJ: **The UCSC Table Browser data retrieval tool.** *Nucleic Acids Res* 2004, **32(Database issue)**:D493-496.

S2. Kuhn RM, Karolchik D, Zweig AS, Wang T, Smith KE, Rosenbloom KR, Rhead B, Raney BJ, Pohl A, Pheasant M, Meyer L, Hsu F, Hinrichs AS, Harte RA, Giardine B, Fujita P, Diekhans M, Dreszer T, Clawson H, Barber GP, Haussler D, Kent WJ: **The UCSC Genome Browser Database: update 2009**. *Nucleic Acids Res* 2009, **37(Database issue)**:D755-761.

**XXyac-R12DG2.2**


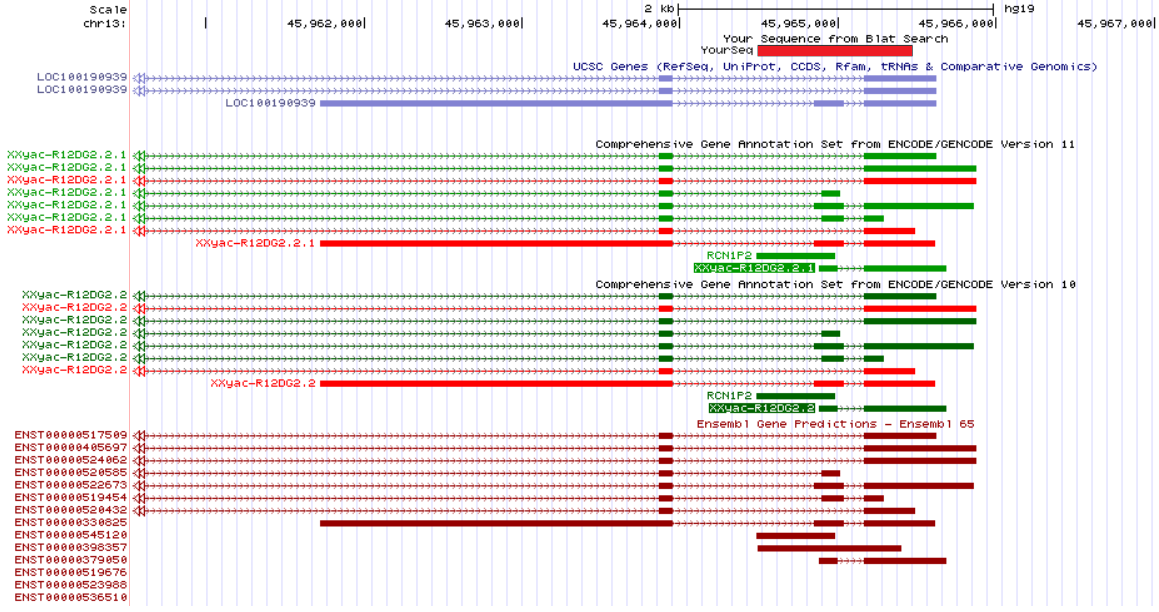


**CSMD3**


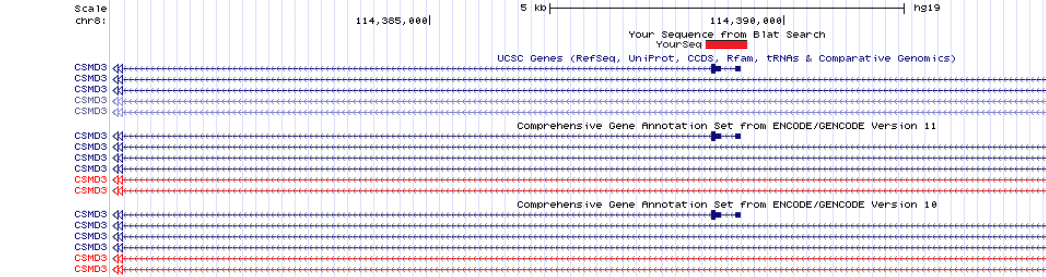


**WBP2NL**


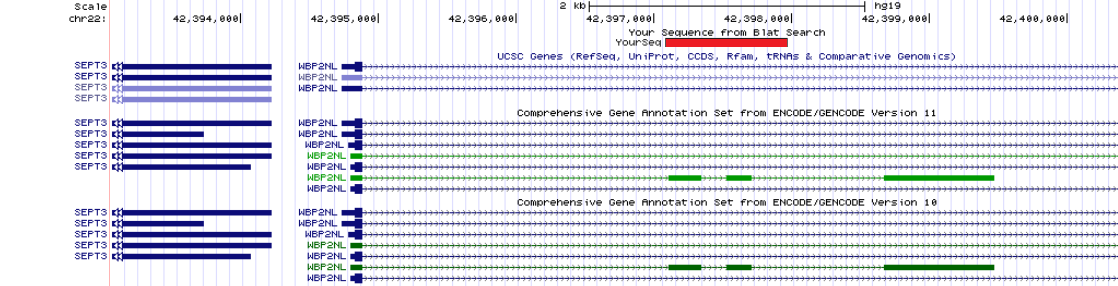

Supplement: Additional file 12 — Positions of three retrogenes (XXyac-R12DG2.2, CSMD3 and WBP2NL) in the human genome (from the UCSC Genome Browser database). This file contains snapshots from the UCSC Genome Browser Database displaying the positions of three retrogenes [file 1471-2148-12-128-S12.doc]
