# Supplementary material for: Newly evolved introns in human retrogenes provide novel insights into their evolutionary roles
Source: BMC Evol Biol. 2012 Jul 28;12:128. doi: 10.1186/1471-2148-12-128 (PMC3565874; doi:10.1186/1471-2148-12-128)
Supplement: Additional file 13 — Comparison of splicing signals (percentile score) in the corresponding region of the new intron in the parental gene and neighboring introns. This file shows the results for the comparison of splicing signals in the corresponding region of the new intron in the parental gene and neighboring introns [file 1471-2148-12-128-S13.doc]

**Additional file 13**

**Comparison of splicing signals (percentile score) in the corresponding region of the new intron in the parental gene and neighboring introns.**

| Intron Symbol | Target | BS (K) | PPT (K) | 3' SS (M) | 5' SS (M) |
| --- | --- | --- | --- | --- | --- |
| TMEM14D | Intronized region in the retrogene | 0.14 | 0.06 | 0.02 | 0.02 |
|  | The corresponding region of the new intron in the parental gene | 0.14 | 0.04 | 0.11 | 0.01 |
|  | Neighboring two introns of the corresponding region of the new intron in the parental gene | 0.03 | 0.66 | 0.76 | 0.66 |
|  |  | 0.84 | 0.87 | 0.57 | 0.3 |
| HSP90B2P-2 | Intronized region in the retrogene | 0.5 | 0.03 | 0.01 | 0 |
|  | The corresponding region of the new intron in the parental gene | 0.5 | 0.02 | 0.02 | 0 |
|  | Neighboring two introns of the corresponding region of the new intron in the parental gene | 0.56 | 0.29 | 0.65 | 0.23 |
|  |  | 0.45 | 0.01 | 0.27 | 0.3 |

“BS”, branch site. “PPT”, polypyrimidine tract. “SS”, splice site. The higher the score, the stronger the splicing signal is. The scores of BS and PPT are calculated in the algorithm ‘K’ [S1] while that of 5' SS and 3' SS are in ‘M’ [S2] by SROOGLE [S3].

**References**

S1. Kol G, Lev-Maor G, Ast G: **Human-mouse comparative analysis reveals that branch-site plasticity contributes to splicing regulation**. [*Hum Mol Genet*](http://www.ncbi.nlm.nih.gov/pubmed?term=Human-mouse comparative analysis reveals that branch-site plasticity contributes to splicing regulation) 2005, **14(11)**:1559-1568.

S2. Schwartz SH, Silva J, Burstein D, Pupko T, Eyras E, Ast G: **Large-scale comparative analysis of splicing signals and their corresponding splicing factors in eukaryotes**. *Genome Res* 2008, **18(1)**:88-103.

S3. Schwartz S, Hall E, Ast G: **SROOGLE: webserver for integrative, user-friendly visualization of splicing signals**. [*Nucleic Acids Res*](http://www.ncbi.nlm.nih.gov/pubmed?term=SROOGLE%3A webserver for integrative%2C user-friendly visualization of splicing signals) 2009, **37(Web Server issue)**:W189-192.
